# Genome-Wide Analysis of Genetic Variations and the Detection of Rich Variants of NBS-LRR Encoding Genes in Common Wild Rice Lines

**DOI:** 10.1007/s11105-018-1103-1

**Published:** 2018-07-22

**Authors:** Hang Yu, Muhammad Qasim Shahid, Rongbai Li, Wei Li, Wen Liu, Fozia Ghouri, Xiangdong Liu

**Affiliations:** 1 State Key Laboratory for Conservation and Utilization of Subtropical Agro-Bioresources, South China Agricultural University, Guangzhou, 510642 China; 2 State Key Laboratory for Conservation and Utilization of Subtropical Agro-Bioresources, Guangxi University, Nanning, 530004 China; 3 College of Agronomy, Guangdong Ocean University, Zhanjiang, 524000 China; 4 Department of Tropical Crops, Guangdong Agriculture Industry Business Polytechnic College, Guangzhou, 510507 China

**Keywords:** Common wild rice (*Oryza rufipogon* Griff.), Genome re-sequencing, DNA variations, NBS-LRR

## Abstract

Common wild rice (*Oryza rufipogon* Griff.) is invaluable genetic resource for rice resistance breeding. Whole-genome re-sequencing was conducted to systematically analyze the variations in two new inbred lines (Huaye 3 and Huaye 4) developed from a common wild rice. A total of 4,841,127 SNPs, 1,170,479 InDels, 24,080 structural variations (SVs), and 298 copy number variations (CNVs) were identified in three materials. Approximately 16.24 and 5.64% of the total SNPs and InDels of Huaye 3 and Huaye 4 were located in genic regions, respectively. Together, 12,486 and 15,925 large-effect SNPs, and 12,417 and 14,513 large-effect InDels, which affect the integrity of the encoded protein, were identified in Huaye 3 and Huaye 4, respectively. The distribution map of 194 and 245 NBS-LRR encoding homologs was constructed across 12 rice chromosomes. Further, GO enrichment analysis of the homologs with identical genotype variations in Huaye 3 and Huaye 4 revealed 67, 82, and 58 homologs involved in cell death, response to stress, and both terms, respectively. Comparative analysis displayed that 550 out of 652 SNPs and 129 out of 147 InDels were present in a widely used blast-susceptible rice variety (LTH). Protein-protein interaction analysis revealed a strong interaction between NBS-LRR candidates and several known R genes. One homolog of disease resistance protein (RPM1) was involved in the plant-pathogen interaction pathway. Artificial inoculation of disease/insect displayed resistance phenotypes against rice blast and brown planthopper in two lines. The results will provide allele-specific markers for rice molecular breeding.

**Electronic supplementary material:**

The online version of this article (10.1007/s11105-018-1103-1) contains supplementary material, which is available to authorized users.

## Introduction

Rice is one of the most important foods for over half of the world’s population. The increasing world population needs higher rice productivity. However, insects, pests and diseases, and abiotic stresses have become the major restrictions for rice productivity. Some new disease and insect resistance, and stress-tolerant cultivars need to be evolved to meet climate changing and pathogen plant co-evolution. The non-domesticated common wild rice (*Oryza rufipogon* Griff.) has been proved an invaluable resistance genetics resource for rice genetic improvement due to its high level of resistance against various biotic and abiotic stresses (Tanksley and McCouch 1997; Song et al. 2005).

Common wild rice accumulated valuable resistance genes and alleles during long-term exposure to the natural environment. Many insect and disease resistance genes and QTLs have been identified from wild rice lines, such as rice blast resistance genes (*Pi9*, *Pi40(t)*, *Pi-ta*) (Qu et al. 2006; Jeung et al. 2007; Geng et al. 2008) and brown planthopper (BPH) resistance genes (*Bph10(t)*, *Bph18(t)*, *Bph14*) (Ishii et al. 1994; Jena et al. 2006; Du et al. 2009). Most of the cloned resistance genes (R genes) in rice are nucleotide-binding leucine-rich repeats (NBS-LRR) that involved in effector-triggered immunity (ETI) (Dangl et al. 1996). The host plant will get systemic acquired resistance (SAR) and significantly enhance the resistant after R protein regulated programmed cell death (PCD) at the site of infection during hypersensitive response and the ETI process (Fu and Dong 2013).

The achievements of the Nipponbare complete genome sequence by the International Rice Genome Sequencing Project (2005) provided a well-assembled reference genome for next-generation re-sequencing (NGR) technologies (Feuillet et al. 2011; Gao et al. 2012). NGR technologies largely simplified the process of DNA variation detection, made it easier to reveal the genetic diversity among different accessions, and provided opportunity to understand the correlation between the DNA polymorphisms and phenotype differentiation. Genome-wide DNA variations include single-nucleotide polymorphism (SNP), insertion/deletion (InDel), and large segment variations, such as structural variation (SV) and copy number variation (CNV) (Varshney et al. 2009; Huang et al. 2013). These DNA variations are responsible for the phenotype divergence among different rice germplasm. The abundant SNPs in the genome can be used for the determination of population structure, the measurement of linkage disequilibrium, and the bulked segregation analysis (Huang and Han 2014; Candela et al. 2015). InDels have been used for development of genome-wide markers for the specified phenotype (Hayashi et al. 2006; Liu et al. 2015; Yonemaru et al. 2015). SVs consist of large-scale insertions, deletions, and translocations, which are usually considered to be related with gain/loss of genes. CNVs revealed copy number gained and copy number loss in more than 1-kb length. These DNA variations present in the regulatory or coding regions can affect the gene expression or function.

Large amount of DNA sequence polymorphisms has been detected in rice and other plants by the whole-genome re-sequencing. The variation database between two Asian rice subspecies was established based on the draft genome of the *japonica* cultivar, Nipponbare, and the *indica* cultivar, 9311 (Feltus et al. 2004; Shen et al. 2004). The low-depth re-sequencing was used to detect the evolutionary relationship among large samples (McNally et al. 2009). High-depth re-sequencing provides a higher accuracy in variation detection and can be used for understanding the genetic basis underlying the divergence, and detecting candidate genes (Subbaiyan et al. 2012). An *indica* restorer line, 7302R, with ideal plant architecture was re-sequenced, and the genomic variants associated with ideal plant architecture were detected (Li et al. 2012). Re-sequencing of two rice accessions with low-phosphate-tolerant and -sensitive phenotypes exhibited several DNA polymorphisms in key phosphate starvation-responsive and root architecture genes (Mehra et al. 2015). Rice cultivars with contrasting drought and salinity stress response were re-sequenced and the variations in drought and salinity response QTLs were explored (Jain et al. 2014). Some NBS-LRR genes had been identified in two newly developed rice lines from common wild rice, which were indigenous to Dongxiang, Jiangxi Province, China, by using genome-wide re-sequencing (Liu et al. 2017). Recently, rice pan-genome data provided the information about thousands of newly identified genes, present/absent variations, and unique genes of cultivated and wild lines (Zhao et al. 2018).

In the present study, we reported two stable inbred lines, Huaye 3 and Huaye 4, with relatively high resistance to rice blast and brown planthopper, developed from a common wild rice, S24, which is indigenous to Suixi, Guangdong Province, China. In order to reveal the genome variation features of two inbred lines and their common wild rice progenitor and to get a preliminary understanding of the genetic basis lies in resistance phenotype, whole-genomes re-sequencing of S24, Huaye 3, and Huaye 4 was performed. We investigated the distribution features of four categories of DNA variations in whole genome, viz., SNPs, InDels, SVs, and CNVs in the three materials, and these variations were functionally annotated to reveal the functional candidates. Moreover, we also constructed a whole-genome variants map of NBS-LRR encoding genes of Huaye 3 and Huaye 4. This study will facilitate the utility of these variations and resistance alleles in molecular breeding and functional genomics.

## Materials and Methods

### Agronomic Traits Observation and Resistance Evaluation of Plant Materials

The common wild rice S24 is conserved at our wild rice germplasm center (South China Agricultural University), and the two inbred lines, Huaye 3 and Huaye 4, derived from S24, were planted at our research farm. Agronomic traits, including plant height, flag leaf length, flag leaf width, main panicle length, productive panicle number, seed setting, ratio of seed length to seed width, grain yield per plant, and 1000-grain weight, of Huaye 3 and Huaye 4 were measured at maturity during 2016 according to the protocols of the People’s Republic of China for the registration of a new plant variety DUS (distinctness, uniformity, and stability) test guidelines of rice (*Oryza sativa* L.) (Guidelines for the DUS test in China 2012; Guo et al. 2017). Single-factor variance analysis was conducted in Microsoft Excel 2010. Ten rice blast isolates, GD09-15, GD09-9, GW1, GW2, GW6, HB7, HN4, HN7, SH10, and SH5, were used to evaluate the blast resistance levels of Huaye 3 and Huaye 4 by single inoculation according to the method described by Mackill (1992). The brown planthopper resistance assay of Huaye 3 and Huaye 4 was conducted according to the method of Wang et al. (2015). The BPH-susceptible *indica* variety, Taichuang Native 1 (TN1), was used as control. When all the susceptible plants (control) died, six resistance levels (0, 1, 3, 5, 7, and 9) were used to score the BPH resistance according to the International Rice Research Institute (IRRI) guidelines (IRRI 1988). Moreover, resistance performance of Huaye 3 and Huaye 4 against BPH was also observed in disease epidemic years of 2015 and 2017.

### Sample Preparation and Sequencing

Young leaves of the common wild rice progenitor S24 and two inbred lines Huaye 3 and Huaye 4 were collected and stored at − 80 °C for DNA extraction. Genomic DNA was extracted using a modified CTAB method (Cota-Sanchez et al. 2006). Genomic DNA quality was evaluated by Nanodrop 2000 and agarose gel electrophoresis. Sequencing library was prepared according to the standard protocol of Illumina. Then, pair-end sequencing was conducted by Illumina HiSeqTM 2500 and Hiseq X Ten platform. The re-sequencing data of LTH were downloaded and transformed to FASTQ format using an SRA Toolkit. The generated FASTQ file quality was evaluated using FastQC (http://www.bioinformatics.babraham.ac.uk/projects/fastqc/). Then, three types of low-quality reads, viz., reads with sequencing adapter, reads with more than 10% N content, and reads with more than 50% low-quality bases (< 10), were filtered.

### Reads Mapping

The filtered high-quality reads were then mapped to the latest Nipponbare reference genome (MSU7.0, http://rice.plantbiology.msu.edu/) using BWA software (Li and Durbin 2009). MarkDuplicates in Picard (https://sourceforge.net/projects/picard/) were used to eliminate the PCR duplication. Base recalibration and realignment near insertion or deletion regions were conducted using a Genome Analysis Toolkit (GATK). Reference genome coverage was estimated using SAMtools (McKenna et al. 2010). Newly identified wild rice unique genes about PAV variations were selected from rice pan-genome data (Zhao et al. 2018). The files were downloaded from Rice Pan Genome website (http://202.127.18.228/RicePanGenome/index.php), and the sequencing reads were mapped onto these gene sequences using BWA software, and the coverage was calculated using SAMtools (McKenna et al. 2010).

### Identification and Analysis of Variations

GATK was further used for the detection of SNPs and InDels after the filtration of alignment results. The following SNPs and InDels were filtered: two or more SNPs in a 5-bp or shorter window, SNPs near (5 bp or less) InDels, and two or more InDels in a 10-bp or shorter window. We further retained the SNPs and InDels with a coverage depth ranged from 11× to 100×. SVs were identified by using the BreakDancer software, and SVs with a coverage depth ranged from 21× to 100× were retained (Chen et al. 2009). CNVs were identified by using the FREEC software (Boeva et al. 2012). All the SNPs and InDels were annotated using the SNPEFF software, and SVs and CNVs were also annotated based on the GFF file of the Nipponbare reference genome (Cingolani et al. 2012). Distribution of these variations was further analyzed and the SNP/InDel-rich/-poor regions with high/low density in a 100-kb window were detected using a five-number summary boxplot.

### Validation of Variations

The CDS sequences of randomly selected variation regions were used as template for primer design. Primer Premier 5 software was used for primer designing, and the primers were validated using NCBI Primer-BLAST (https://www.ncbi.nlm.nih.gov/tools/primer-blast/). Polymerase chain reaction (PCR) was used to amplify the variation regions in a 20-μL volume containing 30 ng template, 0.15 μmol/L primer pairs, 1.0 μL dNTPs (2.0 mmol/L each), one 1 U Taq polymerase, and 1× PCR buffer (50 mmol/L KCl, 10 mmol/L Tris-HCl pH 8.3, 1.5 mmol/L MgCl_2_, 0.01% glutin). The PCR procedure was 94 °C for 5 min followed by 30 cycles of 94 °C for 45 s, 55 °C for 45 s, and 72 °C for 50 s, and a final extension at 72 °C for 5 min. PCR products were examined by agarose gel electrophoresis and further sequenced by Sanger sequencing method. The Sanger sequencing results were further assembled by using DNAMAN software. The assembled sequences were aligned to the reference genome sequences to validate the variations in all the selected regions using ClustalW software.

### Annotation and Functional Prediction of Variant Genes

All the variant gene functions were annotated by gene ontology (GO), Swissprot protein database, Kyoto Encyclopedia of Genes and Genomes (KEGG), Clusters of Orthologous Groups (COG) of protein database, and NCBI non-redundant (NR) database using BLAST software (Altschul et al. 1997). Pfam database annotation was conducted using PfamScan (Li et al. 2015). We further analyzed the gene distribution across different chromosomes of all the annotated NBS-LRR genes in the two inbred lines by using MapDraw V2.1 software (Liu and Meng 2003). NBS-LRR gene clusters were identified based on the former definition of gene cluster in *Arabidopsis* (Holub 2001). GO enrichment analysis was conducted using agriGO (Du et al. 2010). RepeatMasker (http://www.repeatmasker.org/) was used to classify the transposon elements (TEs) in structure variations and copy number variations. The protein-protein interaction network was predicted using the STRING website tool (Szklarczyk et al. 2015).

### Phylogenetic Relationship and Gene Structure Analysis of Candidate Genes and Homologs in Huaye 3 and Huaye 4

The homologs of candidate genes in the two inbred lines were obtained using SAMtools and VCFtools (https://github.com/vcftools/vcftools) based on the Nipponbare reference genome and the VCF files. All the nucleotide sequences were translated to amino acid sequences by using a rice ORF find program, Beijing Gene Finder website tool (BGF, http://bgf.genomics.org.cn/). MEME_4.11.3 program was used to identify the conserved ten motifs (Bailey et al. 2009). Further, all the conserved domains were identified by using NCBI CD-search (https://www.ncbi.nlm.nih.gov/cdd). Multiple sequence alignment was completed using ClustalW software, and neighbor-joining phylogenetic tree was constructed using MEGA7 software with 1000 bootstrap replications (Kumar et al. 2016).

### Data Availability

Re-sequencing raw data have been deposited to the National Center for Biotechnology Information (NCBI) SRA database, and the accession number is “PRJNA396096.” The re-sequencing data of LTH were downloaded from the SRA database (accession numbers: ERX1442095, ERX1442095, and ERX1442095).

## Results

### Breeding Procedure of Huaye 3 and Huaye 4 Developed from Common Wild Rice

**Fig. 1:**
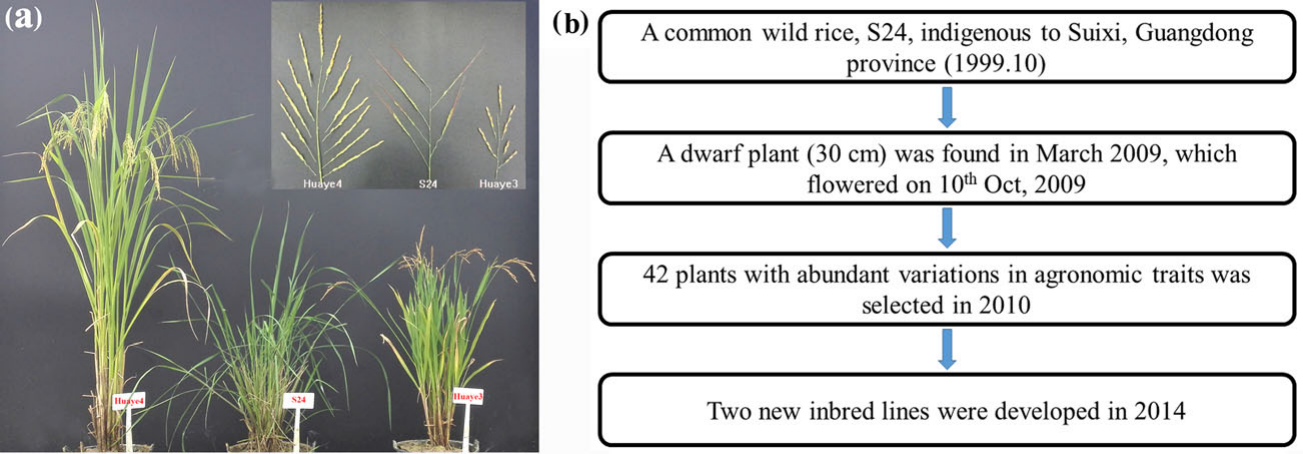
Phenotype and breeding procedure of common wild rice (S24) and its inbred lines (Huaye 3 and Huaye 4). (a) Whole-plant and panicle morphology of common wild rice (S24) and its inbred lines (Huaye 3 and Huaye 4) in the field. (b) Breeding procedure of Huaye 3 and Huaye 4

Two new inbred lines were developed from a common wild rice (S24) by our research group in 2014, which were designated as “Huaye 3” and “Huaye 4” in 2016 (Fig. S1). These two rice lines displayed significant differences in agronomic traits, including plant height, ratio of flag leaf length to width, main panicle length, productive panicle number, seed setting, ratio of seed length to width, grain yield per plant, and 1000-grain weight (Fig. 1, Table 1). The plant height and panicle number of Huaye 3 were 60.24 cm and 15.08, while Huaye 4 displayed 121.15 cm and 6.65, respectively (Table 1).

**Table 1:** Agronomic traits data of Huaye 3 and Huaye 4 during 2016

Agronomic traits	Early season			Late season		
	Huaye 3 (average ± SE)	Huaye 4 (average ± SE)		Huaye 3 (average ± SE)	Huaye 4 (average ± SE)	
Plant height (cm)	64.23 ± 2.29	126.10 ± 6.38	**	56.24 ± 2.80	116.20 ± 2.90	**
Flag leaf length (cm)	12.52 ± 1.27	34.06 ± 4.70	**	14.44 ± 2.05	46.16 ± 6.94	**
Flag leaf width (cm)	1.24 ± 0.10	1.53 ± 0.10	**	1.14 ± 0.11	1.76 ± 0.07	**
Ratio of flag leaf length to width	10.10 ± 0.62	22.40 ± 3.70	**	12.66 ± 1.76	26.18 ± 3.78	**
Main panicle length (cm)	13.75 ± 0.65	27.69 ± 1.78	**	13.21 ± 1.05	29.33 ± 1.65	**
Productive panicle number	14.85 ± 2.58	5.80 ± 0.95	**	15.30 ± 6.42	7.50 ± 2.40	**
Seed setting (%)	89.43 ± 5.19	57.23 ± 5.95	**	80.07 ± 5.38	76.41 ± 4.01	*
Ten-grain length (cm)	7.55 ± 0.12	8.68 ± 0.26	**	7.20 ± 0.08	8.27 ± 0.12	**
Ten-grain width (cm)	2.60 ± 0.06	2.65 ± 0.08	*	2.17 ± 0.06	2.03 ± 0.04	**
Ratio of grain length to width	2.90 ± 0.09	3.27 ± 0.13	**	3.32 ± 0.09	4.08 ± 0.08	**
Grain yield per plant (g)	4.65 ± 1.11	7.36 ± 2.59	**	3.21 ± 1.19	16.79 ± 5.16	**
1000-grain weight (g)	13.19 ± 1.51	15.42 ± 1.87	**	13.35 ± 0.76	16.75 ± 0.41	**

“*” represent significant difference (*P* < 0.05); “**” represent highly significant difference (*P* < 0.01)

### Whole-Genome Re-sequencing and Reads Mapping

Whole-genome re-sequencing of S24, Huaye 3, and Huaye 4 generated 79,619,359, 95,853,301, and 77,152,550 raw reads, and about 97.85, 99.58, and 97.54% of these were high-quality reads. About 89.60, 87.98, and 86.85% of these bases were high-quality bases (quality score ≧ Q30). The G/C contents of S24, Huaye 3, and Huaye 4 were 43.46, 44.97, and 41.43%, respectively. The high-quality reads were further mapped onto the Nipponbare reference genome (MSU7.0) uniformly using BWA software (Fig. S2). Overall, 89.76, 90.34, and 91.17% of these reads were uniquely mapped and covered 91.71, 91.47, and 87.52% of reference genome at 10× coverage depth, and the average coverage depth was 44×, 65×, and 42× in S24, Huaye 3, and Huaye 4, respectively (Table 2).

**Table 2:** Summary of re-sequencing data and reads mapping onto the Nipponbare genome

	S24	Huaye 3	Huaye 4
Total reads	79,619,359	95,853,301	77,152,550
High-quality reads	77,907,543	95,453,868	75,256,129
Clean bases	19,629,347,386	28,611,001,160	18,960,007,854
Percent of bases ≥ Q20 (%)	96.55	94.67	92.47
Percent of bases ≥ Q30 (%)	89.6	87.98	86.85
Mapped (%)	96.54	97.71	97.83
Properly mapped (%)	89.76	90.34	91.17
Coverage ratio 10× (%)	91.71	91.47	87.52
GC content (%)	43.46	44.97	41.43
Average coverage depth	44	65	42

### Detection and Validation of DNA Polymorphisms

A total of 2,018,840 (1,804,889 SNPs and 213,951 InDels), 1,505,317 (1,349,511 SNPs and 155,806 InDels), and 1,867,413 (1,686,727 SNPs and 180,686 InDels) DNA polymorphic sites were identified in S24, Huaye 3, and Huaye 4 compared to the Nipponbare reference genome, respectively (Tables 3 and 4). Venn analysis indicated that Huaye 3 and Huaye 4 shared more identical DNA polymorphic sites than S24/Huaye 3 and S24/Huaye 4 (Fig. S3a).

**Table 3:** Number of total SNPs and different types of SNP variations

Material	SNP number	Transition (Ti)	Transversion (Tv)	Ti/Tv	Heterozygosity (Het)	Homozygosity (Hom)	Het percentage
S24	1,804,889	1,274,152	530,737	2.4	987,563	817,326	54.71
Huaye 3	1,349,511	964,498	385,013	2.5	157,094	1,192,417	11.64
Huaye 4	1,686,727	1,209,376	477,351	2.53	191,451	1,495,276	11.35

**Table 4:** Number of insertions and deletions in whole genome and coding regions

Material	Insertions genome (CDS, percentage)	Deletions genome (CDS, percentage)	Total genome (CDS, percentage)
S24	213,951 (12,373, 5.78)	231,017 (13,472, 5.83)	444,968 (25,845, 5.81)
Huaye 3	155,806 (8665, 5.56)	178,108 (10,126, 5.69)	333,914 (18,791, 5.63)
Huaye 4	180,686 (9925, 5.49)	210,911 (11,769, 5.58)	391,597 (21,694, 5.54)

In order to evaluate the accuracy of the variations detected by re-sequencing, 18, 13, and 13 genomic regions with abundant DNA variations in S24, Huaye 3, and Huaye 4 respectively were randomly selected for variation validation using Sanger sequencing. In total, 114, 88, and 85 DNA variation sites were validated in S24, Huaye 3, and Huaye 4, respectively. The results showed that the DNA variation sites detected by using Sanger sequencing were consistent with the re-sequencing data (Table S1).

### Distribution of SNPs and InDels

The number and density distribution of DNA polymorphisms varied across different chromosomes. The highest SNP numbers were observed on Chr1, while Chr9, Chr12, and Chr8 had the lowest number of SNPs in S24, Huaye 3, and Huaye 4, respectively (Fig. S4a). Similarly, the highest InDel numbers were observed on Chr1, while Chr9, Chr9, and Chr8 had the lowest number of InDels in S24, Huaye 3, and Huaye 4, respectively (Fig. S4b). The highest SNP and InDel densities were found on Chr11, Chr10, and Chr10, and the lowest SNP and InDel densities were found on Chr5, Chr12, and Chr8 in S24, Huaye 3, and Huaye 4, respectively (Fig. S4c, d).

The average SNP densities were 483.57, 361.56, and 451.91 SNP/100 kb, and the average InDel densities were 119.01, 89.31, and 104.75 InDel/100 kb in S24, Huaye 3, and Huaye 4, respectively. However, the SNPs and InDels were not uniformly distributed along the chromosomes (Fig. S5). DNA polymorphism number in every 100-kb window represents the SNP and InDel density (number per 100 kb) along the chromosomes. Variation-rich and variation-poor regions were detected along the 12 rice chromosomes using a five-number boxplot. The outliers of the boxplot were considered as the variation-rich or variation-poor regions (Fig. S6). More variation-rich regions were detected than variation-poor regions in the three materials, and more variation-rich regions in S24 than in Huaye 3 and Huaye 4. In S24, Huaye 3, and Huaye 4, a total of 59, 14, and 23 SNP-rich regions and 178, 50, and 67 InDel-rich regions were detected. However, only 1, 10, and 37 SNP-poor regions and 1, 0, and 30 InDel-poor regions were detected (Table S2). Moreover, nine variation-rich regions (Chr01:12.5–12.6, Chr12:27.0–27.1, Chr10:20.3–20.4, Chr10:2.9–3.0, Chr09:13.9–14.0, Chr12:22.4–22.5, Chr05:19.4–19.5, Chr10:15.9–16.0, and Chr06:4.3–4.4) were jointly shared by S24, Huaye 3, and Huaye 4. These regions may contain the sequence with fast evolution speed between common wild rice and modern cultivated rice.

SNPs are abundant markers distributed across the whole genome, so the SNP zygosity can represent genome zygosity. Analysis on the distribution of SNP zygosity across the 12 rice chromosomes indicated that the wild rice progenitor S24 genome contained much more heterozygous SNPs than the two inbred lines on each chromosome (Table S3). In S24, the proportion of heterozygous SNPs on each chromosome ranged from 47.40 to 68.02%, and the average value was 54.75%. In Huaye 3 and Huaye 4, the proportion of heterozygous SNPs ranged from 8.10 to 25.47%, and the average values were 11.61 and 11.38%, respectively.

### Analysis and Annotation of SNPs and InDels

Investigation on the nucleotide substitution type of SNPs indicated higher frequency of transitions (C/T and G/A, Ti) than transversions (C/A, G/T, C/G, and T/A, Tv), and the ratios of Ti to Tv were 2.40, 2.50, and 2.53 in S24, Huaye 3, and Huaye 4, respectively (Table 2). The frequency of G/C was lower than the other three types of Tv (Fig. S3b). The number of deletions was slightly higher than that on insertions, and only 5.5% of these InDels were in the CDS regions (Table 3). About 46.22, 46.28, and 47.05% of the total InDels were single-nucleotide InDels in S24, Huaye 3, and Huaye 4, respectively. Most of the InDels (91.00% in S24, 90.29% in Huaye 3, 90.21% in Huaye 4) ranged from 1 to 9 bp. Interestingly, we detected a relatively higher frequency of triple-nucleotide InDels in the CDS regions, almost 44.98, 43.85, and 42.74% InDels were triple-nucleotide in S24, Huaye 3, and Huaye 4, respectively (Fig. S3c).

SNPs and InDels of S24, Huaye 3, and Huaye 4 showed a similar distribution in different genomic regions. Approximately 64% SNPs and 70% InDels were distributed in the upstream regions (UPSTREAM, 2 kb), intergenic regions (INTERGENIC), and downstream regions (DOWNSTREAM, 2 kb). About 36% SNPs and 30% InDels were observed in the genic regions, and 668,533, 487,884, and 599,574 SNPs and 136,396, 98,221, and 113,176 InDels were detected in genic regions of S24, Huaye 3, and Huaye 4, respectively. The SNPs and InDels, which were distributed in the initiation/termination codon (START_LOST, NON_SYNONYMOUS_START, STOP_GAINED and STOP_LOST) and splice site regions (SPLICE_SITE_REGION, SPLICE_SITE_ACCEPTOR and SPLICE_SITE_DONOR), and the InDels, which cause frameshift (FRAME_SHIFT), were defined as the large-effect variations that have severe impact on the integrity of encoded products and the gene function. A total of 17,277, 12,486, and 15,925 large-effect SNPs, and 16,816, 12,417, and 14,513 large-effect InDels were identified spanning 16,692, 12,248, and 14,434 variant genes in S24, Huaye 3, and Huaye 4, respectively (Tables S4 and S5).

### Detection and Functional Prediction of SVs and CNVs

**Fig. 2:**
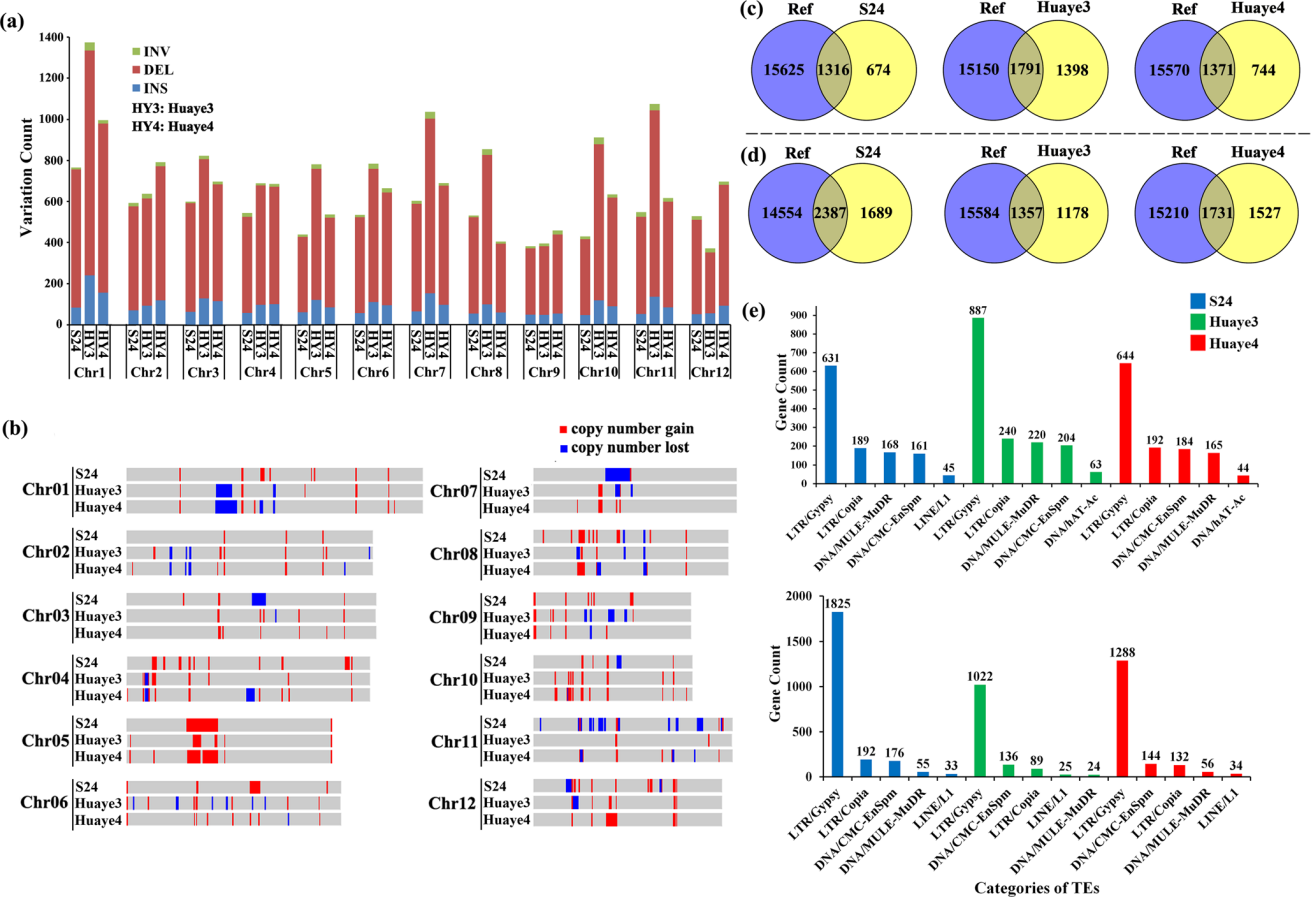
Distribution and annotation of structural variations (SVs) and copy number variations (CNVs). Distribution of different types of SVs (a) and CNVs (b) across 12 rice chromosomes. Venn analysis of SV (c) and CNV (d) variant genes and rice genome transposon elements (TEs). Ref represents the TEs in the Nipponbare genome annotated by International Rice Genome Sequencing Project (2005). Classification of SV (e) and CNV (f) variant TEs

Large segment variations, such as structural variations (SVs) and copy number variations (CNVs), can affect the genome stability. Numerous SVs and CNVs were identified in the three materials. In the intergenic, exon, and intron regions, 6489, 9728, and 7863 SVs in S24, Huaye 3, and Huaye 4 were detected, respectively. Among these SVs, 2055, 3403, and 2169 were in the exon regions (Table S6). Moreover, 70, 75, and 91 copy number gained variations (CNGV) and 20, 24, and 18 copy number loss variations (CNLV) were identified in S24, Huaye 3, and Huaye 4, respectively (Table S7). Three types of SVs, viz., insertion (INS), deletion (DEL), and inversion (INV), were distributed across 12 chromosomes. The number of DELs was much more than INSs and INVs. The maximum SV number was detected on Chr1 in all the three materials, and the minimum SV number was detected on Chr9, Chr12, and Chr8 in S24, Huaye 3, and Huaye 4, respectively (Fig. 2a). Two types of CNVs were distributed across the 12 chromosomes, but only CNGVs were detected on Chr5 in all the three lines. The CNV length was different, and it ranged from 50 Kb to 4.15 Mb, 50 Kb to 2.35 Mb, and 50 Kb to 3.05 Mb in S24, Huaye 3, and Huaye 4, respectively. The longest CNGV (4.15 Mb) and CNLV (3.60 Mb) were detected on Chr05 of S24 (Fig. 2b).

Venn analysis of all Nipponbare transposon elements (TEs) and SV/CNV variant genes indicated that more than half of these genes were TEs (Fig. 2c, d). These SV/CNV TEs were classified by RepeatMasker. Majority of these SV/CNV TEs were long terminal repeat (LTR) retrotransposons, and the number of LTR/Gypsy class was higher than that of other classes (Fig. 2e, f). GO enrichment analysis of the SV/CNV TEs shared by Huaye 3 and Huaye 4 exhibited some transposition or chromatin organization enriched GO terms, such as transposase activity (GO:0004803), chromatin assembly or disassembly (GO:0006333), chromatin organization (GO:0006325), and chromosome organization (GO:0051276) (Tables S8 and S9).

In order to identify the useful genomic regions that were specific to common wild rice lines, we compared our results to the newly released rice pan-genome data (Zhao et al. 2018). A total of 4001 unique genes related to common wild rice were selected and the sequencing reads of S24, Huaye3 and Huaye 4 were mapped onto these genes. The unique genes that fully covered by sequencing reads were considered as wild rice-specific homologs of S24, Huaye 3, and Huaye 4, which were lost from cultivated rice during selection and domestication. In total, 1302, 1118, and 994 wild rice unique homologs were detected in S24, Huaye 3, and Huaye 4 (Table S10), and 725 wild rice unique homologs were shared by the three materials, respectively (Fig. S7).

### Detection of Genome-Wide NBS-LRR Variants in Huaye 3 and Huaye 4

**Fig. 3:**
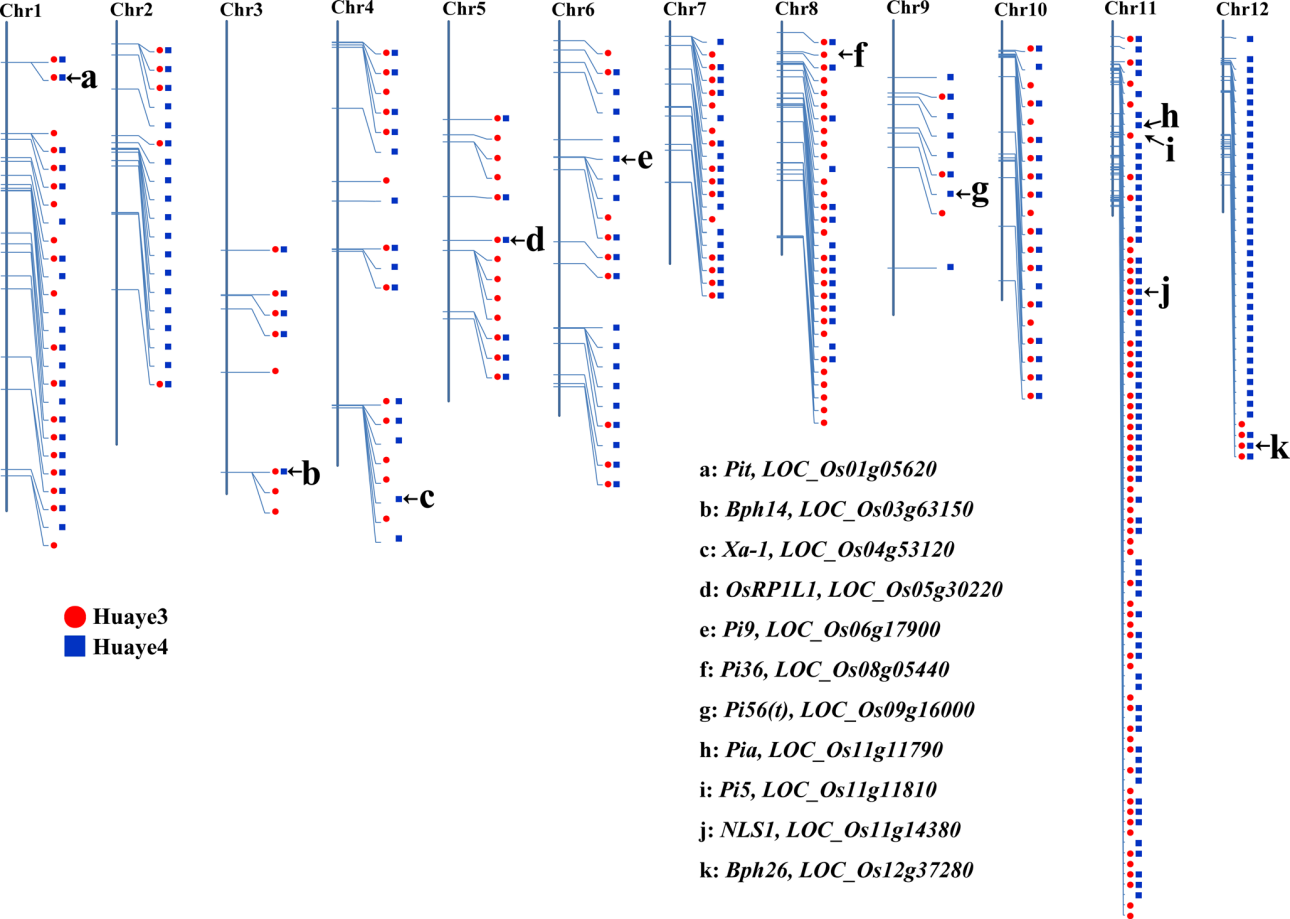
Distribution of annotated NBS-LRR genes in Huaye 3 and Huaye 4. The red dots represent the variant NBS-LRR genes in Huaye 3, and the green boxes represent the variant NBS-LRR genes in Huaye 4. The serial number of homologs in Huaye 3 and Huaye 4 was named as “Or-Chr-NB-18/35-No.” “Or” represents *Oryza rufipogon*, “Chr” represents chromosome, “NB” represents NB-ARC, “18” represents Huaye 3, “35” represents Huaye 4, and “No.” represents serial number

Sequence alignment with the susceptible control Nipponbare detected a total of 194 and 245 NBS-LRR encoding genes with large-effect genetic variations in Huaye 3 and Huaye 4, including six blast resistance genes (*Pit*, *LOC_Os01g05620*; *Pi9*, *LOC_Os06g17900*; *Pi36*, *LOC_Os08g05440*; *Pi56(t)*, *LOC_Os09g16000*; *Pia*, *LOC_Os11g11790*; *Pi5*, *LOC_Os11g11810*), two BPH resistance genes (*Bph14*, *LOC_Os03g63150*; *Bph26*, *LOC_Os12g37280*), two bacterial blight resistance genes (*Xa-1*, *LOC_Os04g53120*; *OsRP1L1*, *LOC_Os05g30220*), and one defense response gene (*NLS1*, *LOC_Os11g14380*) (Table S11). These genes were distributed across the 12 chromosomes. We detected 59 NBS-LRR encoding genes with large-effect variations in Huaye 3 and 67 in Huaye 4 on Chr11, and the maximum number of NBS-LRR variants was located on Chr11. In Chr12, 38 NBS-LRR encoding genes with large-effect variations were detected in Huaye 4, but only four genes were detected in Huaye 3 (Fig. 3). Based on the definition of gene cluster in *Arabidopsis* (Holub 2001), gene clusters (more than three genes in less than 200 kb) of NBS-LRR encoding genes were detected in the present study. In total, 76 of Huaye 3 NBS-LRR variants were grouped into 20 gene clusters, and 101 of Huaye 4 NBS-LRR variants were distributed into 23 gene clusters.

### Functional Enrichment of the Identical Genotype Variants in Huaye 3 and Huaye 4

In order to detect the functional variant genes with identical genotypes in the two inbred lines, similar large-effect SNPs and InDels between Huaye 3 and Huaye 4 were selected for further analysis. A total of 6128 and 4853 identical large-effect SNPs and InDels, which present on 2022 and 3203 variant genes in Huaye 3 and Huaye 4, were identified, respectively.

**Fig. 4:**
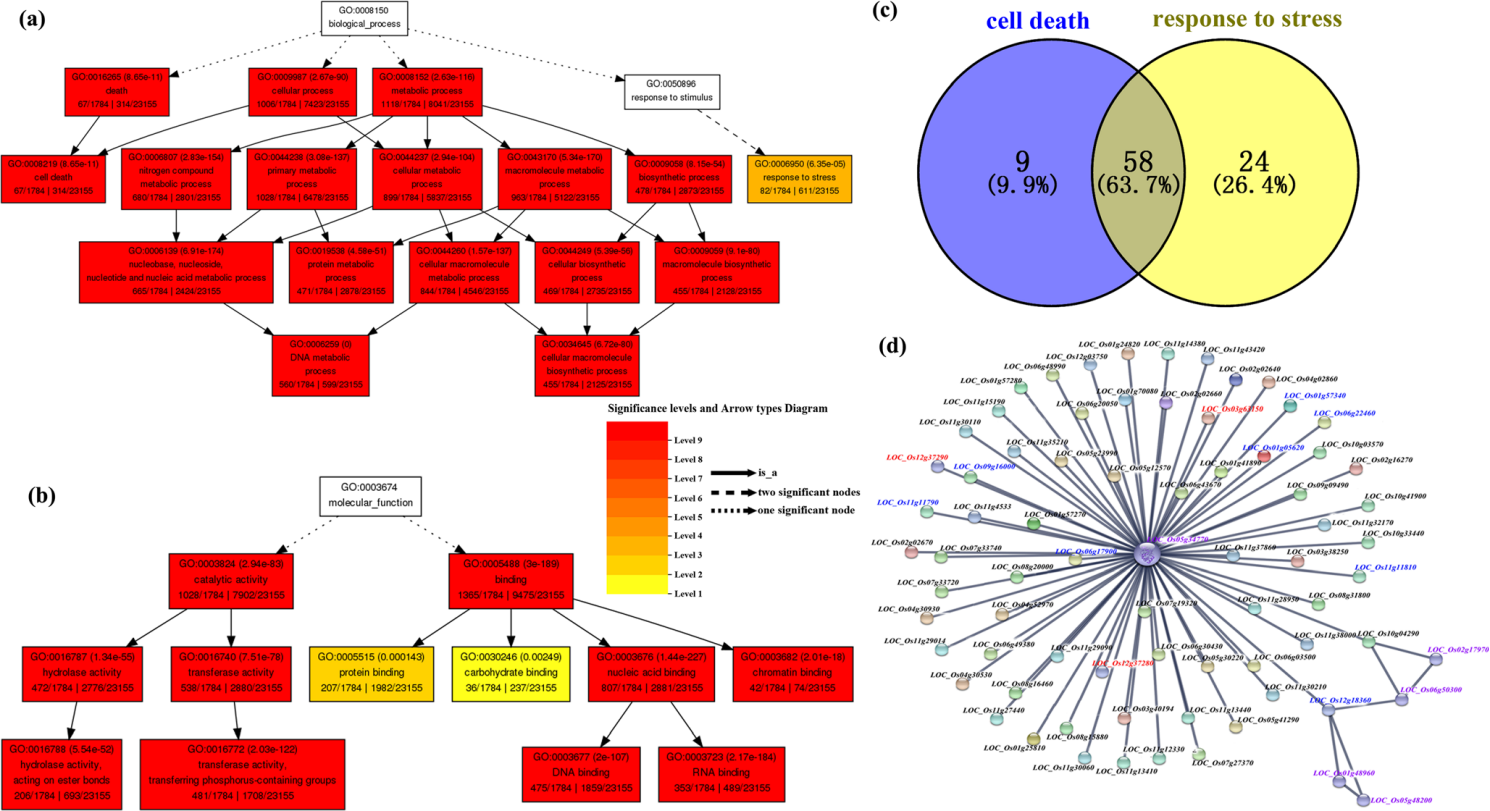
Gene ontology (GO) annotation of the identical SNPs and InDels in Huaye 3 and Huaye 4 and protein interaction network of candidates. (a, b) GO enrichment analysis of identical variant genes in Huaye 3 and Huaye 4. (c) Venn analysis of variant genes involved in two plant resistance response-related GO terms. (d) Predicted protein-protein interaction network of 58 candidate genes and former reported rice resistant genes

The SNPs were distributed in different Pfam domain families, and the ratios of non-synonymous to synonymous SNPs (Ns/Sy SNPs) ranged from 1.00 to 3.67. The ratios of Ns to Sy SNPs in the two conserved domains of NBS-LRR encoding genes NB-ARC and LRR_1 were 2.32 and 2.24, indicating that these two domains have more amino acid substitutions in Huaye 3 and Huaye 4 (Fig. S8). GO enrichment analysis of all the variant genes with similar genotypes were conducted in Huaye 3 and Huaye 4. In biological process category, the genes were enriched in cell death, response to stress, DNA metabolic process, and cellular macromolecule biosynthetic process. In molecular function category, the genes with the function of hydrolase activity, transferase activity, protein binding, carbohydrate binding, nucleic acid binding, and chromatin binding were significantly enriched (Fig. 4a, b). We identified 67 variant genes containing a BPH-resistant gene, *LOC_Os12g37280* (*Bph26*), which were involved in cell death and 82 variant genes were involved in response to stress, while 58 variant genes were involved in both terms (Fig. 4c). All these 91 NBS-LRR homologs contain 652 and 147 homozygous large-effect SNPs and InDels. Comparative analysis indicated that 550 out of 652 SNPs and 129 out of 147 InDels were present in the Chinese widely used blast-susceptible rice variety LTH (Table S12).

These 58 candidate genes were used for predicted protein-protein interaction analysis and revealed a network with eight blast-resistant genes (*Pi-d3*, *LOC_Os06g22460*; *Pi5*, *LOC_Os11g11810*; *Pi56(t)*, *LOC_Os09g16000*; *Pi9*, *LOC_Os06g17900*; *Pia*, *LOC_Os11g11790*; *Pish*, *LOC_Os01g57340*; *Pit*, *LOC_Os01g05620*; *Pita*, *LOC_Os12g18360*) and three BPH-resistant genes (*Bph14*, *LOC_Os03g63150*; *Bph26*, *LOC_Os12g37280*; *BPH18*, *LOC_Os12g37290*). A cytochrome C gene (*LOC_Os05g34770*) connected with the interaction network as a hub gene (Fig. 4d). KEGG pathway annotation revealed a disease resistance protein RPM1 homolog, *LOC_Os09g09490*, which is responsible for the hypersensitive response in plant-pathogen interaction pathway (Fig. S9).

### Phylogenetic and Conserved Motif Analysis of Candidate Homologs in Huaye 3 and Huaye 4

Phylogenetic and MEME analysis of the protein sequences of 58 candidate genes and their homologs in Huaye 3 and Huaye 4 categorized these genes into four groups, including Group I, Group II, Group III, and Group IV (Fig. S10). We used ten motifs to illustrate the conserved region structure of the 174 putative NBS-LRR proteins (Fig. S11), and the details are as follow: 69 proteins were considered as Group I, and most of them were at least related to one NB-ARC domain motif, viz., motif 1, motif 9, or motif 10; 32 proteins were considered as Group II, and most of them were associated with at least one NB-ARC domain motif, viz., motif 1, motif 5, or motif 6; 67 proteins were included in Group III and related with P-loop (motif 4), NB-ARC (motif 6), and three other motifs (motifs 3, 7, 8); and Group IV was comprised of 6 proteins, and all of them were motif 5 and motif 6. The NBS-LRR proteins in different groups may involve in various functions.

NBS-LRR encoding genes and their homologs in Huaye 3 and Huaye 4 were predicted using NCBI CD search to evaluate the variation impact on protein domain structure (Table S13). We found that 28 domain changed NBS-LRR homologs in Huaye 3 and Huaye 4, which may have a significant impact on the gene function and resistant phenotype.

### Preliminary Resistance Evaluation of Huaye 3 and Huaye 4

Artificial resistance inoculation further confirmed the resistance in wild rice lines. Ten races of *Magnaporthe oryzae* inoculation assay revealed that the average blast-resistant level of Huaye 3, Huaye 4, Nipponbare, and LTH were 2.0, 2.7, 3.9, and 5, which indicated higher blast resistance in the two wild rice inbred lines than two control cultivars, respectively. They also showed moderate resistant phenotype for BPH resistance assay, and the resistance scores of Huaye 3 and Huaye 4 were 3 and 5, respectively (Fig. S12, Table S14). Moreover, the resistance phenotypes of the two wild rice inbred lines were observed for 4 years at our farm, and Huaye 3 and Huaye 4 showed high-resistance phenotypes to the occurrence of brown planthopper in the epidemic years of 2015 and 2017.

## Discussion

### Common Wild Rice Lines Possess High DNA Variations

Wild rice showed relatively higher genetic diversity than cultivated rice (Sun et al. 2001, 2002; Tian et al. 2006; Zhu et al. 2007). The high-density DNA polymorphisms obtained by advanced next-generation genome re-sequencing offered us an opportunity to take deeper insights into the whole-genome diversity. The OryzaGenome database provides 11 deeply sequenced common wild rice accessions, and eight of them contained nearly or more than two million SNPs. About 2.5 times higher SNPs were detected in Australian wild rice than Asian wild rice, and *O. sativa* ssp*. indica* compared to Nipponbare reference genome (Krishnan et al. 2014). In the present study, we identified more than 1.5 million polymorphic sites in each material, and all the three samples exhibited higher variation rates compared to Nipponbare reference genome, which are consistent with the previous studies. The high variation rate in the two wild rice inbred lines with low genome heterozygosity rate is also meaningful towards the identification and utilization of favorable alleles.

The occurrence of variation-rich and variation-poor regions has been found in many organisms due to the non-uniform variation distribution (Smith and Lercher 2002; Nordborg et al. 2005; Ravel et al. 2006). This phenomenon may be caused by the artificial selection and selective sweeps during the domestication process of rice (Caicedo et al. 2007). The so-called SNP desert has already been detected in rice cultivars (Wang et al. 2009; Nagasaki et al. 2010), which may represent the existence of highly conserved regions in different rice subspecies (Arai-Kichise et al. 2011). In our study, a total of 201, 60, and 84 variation-rich regions, but only 2, 7, and 47 variation-poor regions were identified in S24, Huaye 3, and Huaye 4, respectively. The presence of variation-rich regions in higher number than that of variation-poor regions indicated a highly divergence and lack of conserved regions between these three common wild rice lines and Asian cultivar Nipponbare.

Large segment DNA variations are also important to genetic diversity and domestication study, especially in wild rice. The common wild rice lost transcripts and Nipponbare acquired genes, which is caused by large segment deletion and insertion in Dongxiang common wild rice (Zhang et al. 2016). Based on the wild rice unique genes of pan-genome data (Zhao et al. 2018), we identified important genes in S24, Huaye 3 and Huaye 4 that were lost from cultivated rice during selection and domestication. In the present study, about 58% of the SV/CNV variant genes are transposable elements (TEs), while the percentage in the Rice Genome Annotation Project was 30% (Kawahara et al. 2013). The high percentage of SV/CNV variant TEs is reported before in mouse genome (Quinlan et al. 2010). The transposons played a key regulatory role in plant stress response (Negi et al. 2016), which are responsible for gene expression change and phenotypic differentiations (Singh et al. 2014; Dhadi et al. 2015; Tan et al. 2017). These SV/CNV variant TEs may cause a dramatic genome rearrangement and have a strong influence on gene expression and resistance level of these three materials.

### Abundant NBS-LRR Homologs May Improve Resistance Through Hypersensitive Response

A comprehensive understanding of the whole-genome variations about resistant genes is beneficial to get full usage of the elite genetic resources. NBS-LRR genes are the largest class of resistant genes in plants, and their variation patterns among different accessions have been studied extensively. A total of 535 NBS-coding genes were found in the Nipponbare genome, one fourth of these genes localized on chromosome 11, and 51% of these genes were in gene clusters (Zhou et al. 2004). Even though numerous resistance genes in rice have been mapped and cloned, and some of these different genes are subsequently proved allelic variations. Zhao et al. (2016) demonstrated that the eight BPH resistance genes on chromosome 12L are allelic with each other. Three rice blast resistance genes, *Pi9*, *Pi2*, and *Piz-t*, have been proved allelic with each other, and they were in one NBS-LRR gene cluster on Chr06 and only have several amino acid differentiations (Zhou et al. 2006). *Pid3* and *Pi25* were in the same locus on Chr06 and only have one amino acid differentiation (Chen et al. 2011). *Pikm*, *Pikh*, *Pik-p*, and *Pi1* are also allelic variations in the same locus on Chr06 (Costanzo and Jia 2010; Ashikawa et al. 2012; Kumari et al. 2013). Therefore, the variation detection of resistance genes in highly resistant rice lines can be a good method to reveal the elite resistance alleles.

A previous study has shown that a single amino acid variation could change *Pi-ta* allele from resistant to susceptible (Bryan et al. 2000). There are abundant structural and genetic variations in different rice accessions, which indicate a highly differentiation in resistance performance (Bai et al. 2002; Yang et al. 2008). Here, investigation on whole-genome variants of NBS-LRR encoding genes of two common wild rice inbred lines with high resistance to rice blast and BPH revealed potentially favorable resistant alleles. A total of 194 and 245 NBS-LRR encoding genes exhibited large-effect variations, including 11 previously cloned R genes, and were explored in Huaye 3 and Huaye 4. Interestingly, only five NBS-LRR encoding genes have intersection with the genes recently identified in another two common wild rice lines (Liu et al. 2017). About 30% of these genes were located on chromosome 11, and 40% of these genes were distributed in gene clusters.

In the process of plant effector-triggered immunity (ETI), the programmed cell death (PCD) following the recognition of pathogen effector (Avr protein) by the R protein is a sign of plant disease resistance. Through PCD in the cell of pathogen infection site, the host plant can get non-specific resistance to other pathogens, known as systemic acquired resistance (SAR). Thus, the host plant can get broad-spectrum resistance (Tang et al. 1999; Spoel and Dong 2012). In the present study, identical SNPs and InDels were obtained in two inbred lines, which reside in 4583 variant genes. GO enrichment analysis indicated that these genes were significantly enriched in cell death, response to stress, hydrolase activity, and nucleic acid binding. A total of 58 annotated NBS-LRR genes involved in cell death and response to stress were considered as potential candidate genes. The gene function further explained by the predicted protein-protein interaction network among eight blast-resistant genes and three BPH-resistant genes. The variant alleles of these 58 candidate NBS-LRR genes were considered as main factors for highly resistant phenotypes in Huaye 3 and Huaye 4. The functional SNPs and InDels detected in our resistant materials could be used as molecular markers for the improvement of elite rice cultivars.

## Electronic supplementary material

ESM 1 (PDF 4873 kb)

ESM 2 (XLSX 353 kb)
