# Supplementary material for: Genome-Wide Analysis of Genetic Variations and the Detection of Rich Variants of NBS-LRR Encoding Genes in Common Wild Rice Lines
Source: Plant Mol Biol Report. 2018 Jul 22;36(4):618–30. doi: 10.1007/s11105-018-1103-1 (PMC6182389; doi:10.1007/s11105-018-1103-1)
Supplement: Supplementary file 1 — (PDF 4873 kb) [file 11105_2018_1103_MOESM1_ESM.pdf]

Supplementary Materials: Figures

Genome-wide analysis of genetic variations and the detection of rich variants of NBS-LRR encoding genes in common wild rice lines

Hang Yu[1], Muhammad Qasim Shahid[1**], Rongbai Li[2], Wei Li[3], Wen Liu[1,4], Fozia Ghouri[1], Xiangdong Liu[1*]**

[1] State Key Laboratory for Conservation and Utilization of Subtropical Agro-Bioresources, South China Agricultural University, Guangzhou 510642, China

[2] State Key Laboratory for Conservation and Utilization of Subtropical Agro-Bioresources, Guangxi University, Nanning 530004, China

[3] College of Agronomy, Guangdong Ocean University, Zhanjiang 524000, China

[4] Department of Tropical Crops, Guangdong Agriculture Industry Business Polytechnic College, Guangzhou 510507, China

*Corresponding authors: Xiangdong Liu Tel.: +86 20 85280205 Fax: +86 20 85280205

E-mail address: xdliu@scau.edu.cn

[**]These authors contributed equally to this work

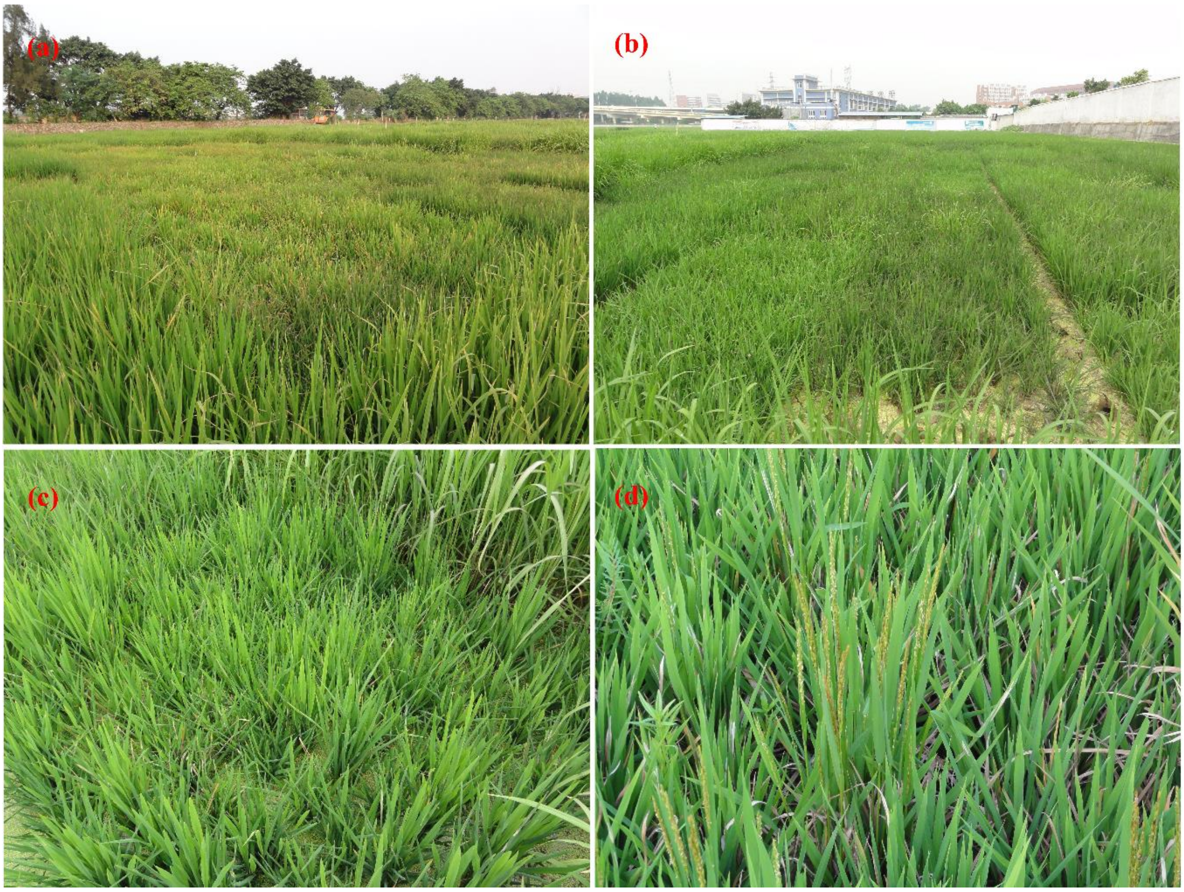

Fig.S1 Inbred lines derived from S24 common wild rice and segregation in thier traits.

(a, b, c) 4th generation of inbred lines derived from S24 grown in the field during early season of 2011. (d) Plants of 4th generation segregation population of S24 at heading stage.

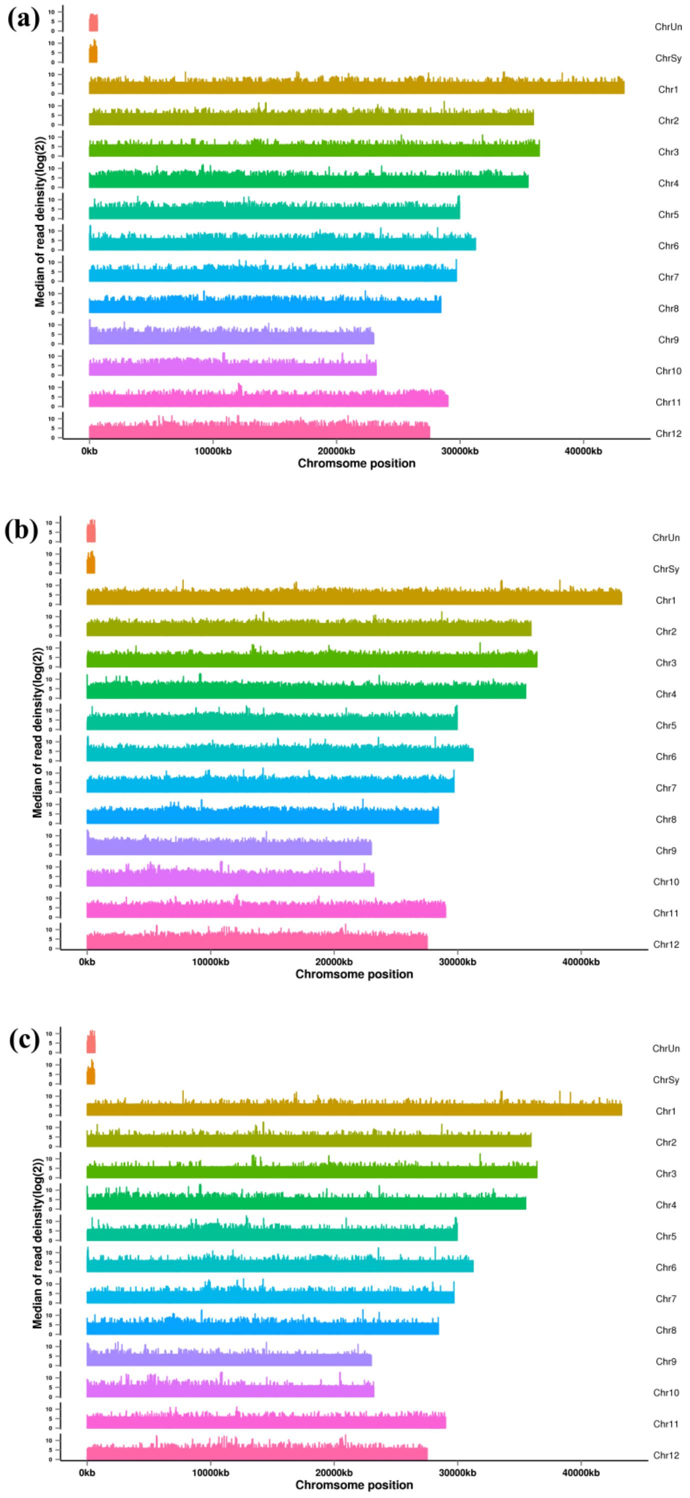

Fig.S2 Genome-wide distribution of reads mapping coverage in S24 (a), Huaye 3 (b) and Huaye 4 (c)

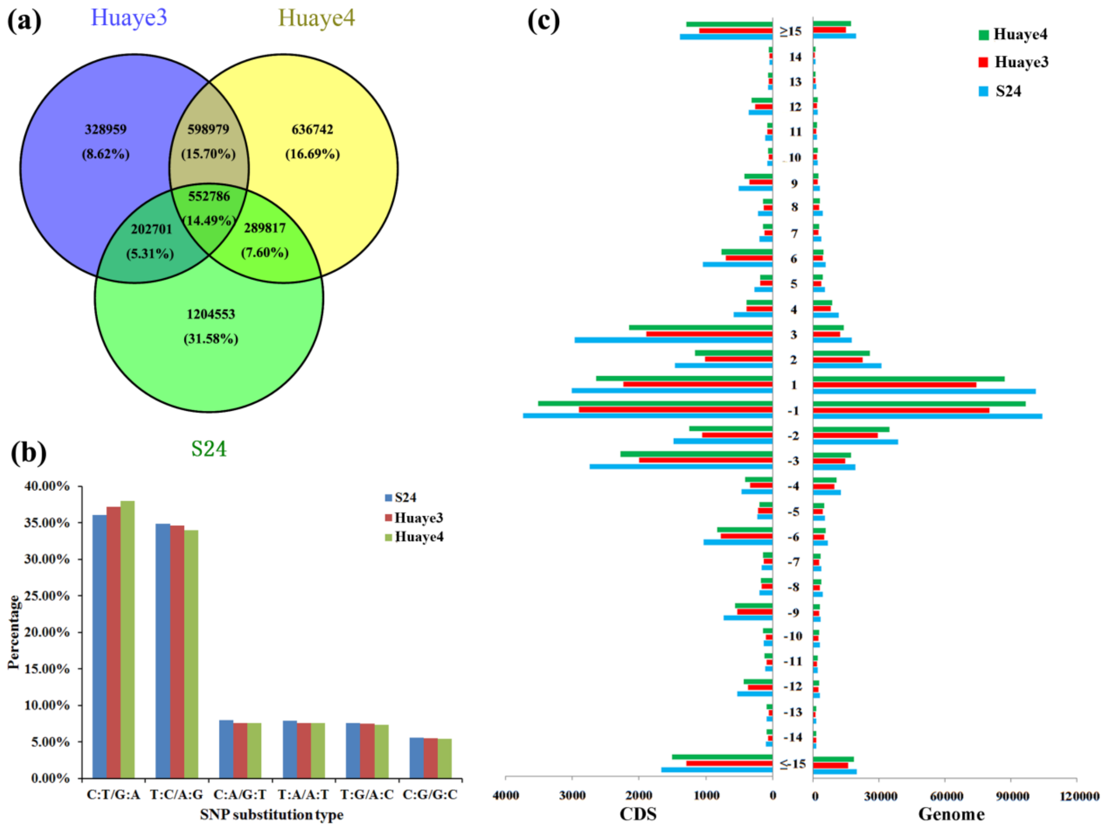

Fig.S3 Distribution of single nucleotide polymorphisms (SNPs) and insertion and deletions (InDels).

(a) Venn analysis of SNPs and InDels in S24, Huaye 3 and Huaye 4. (b) Frequency of six substitution types of SNPs. (c) Distribution of InDels in different length.

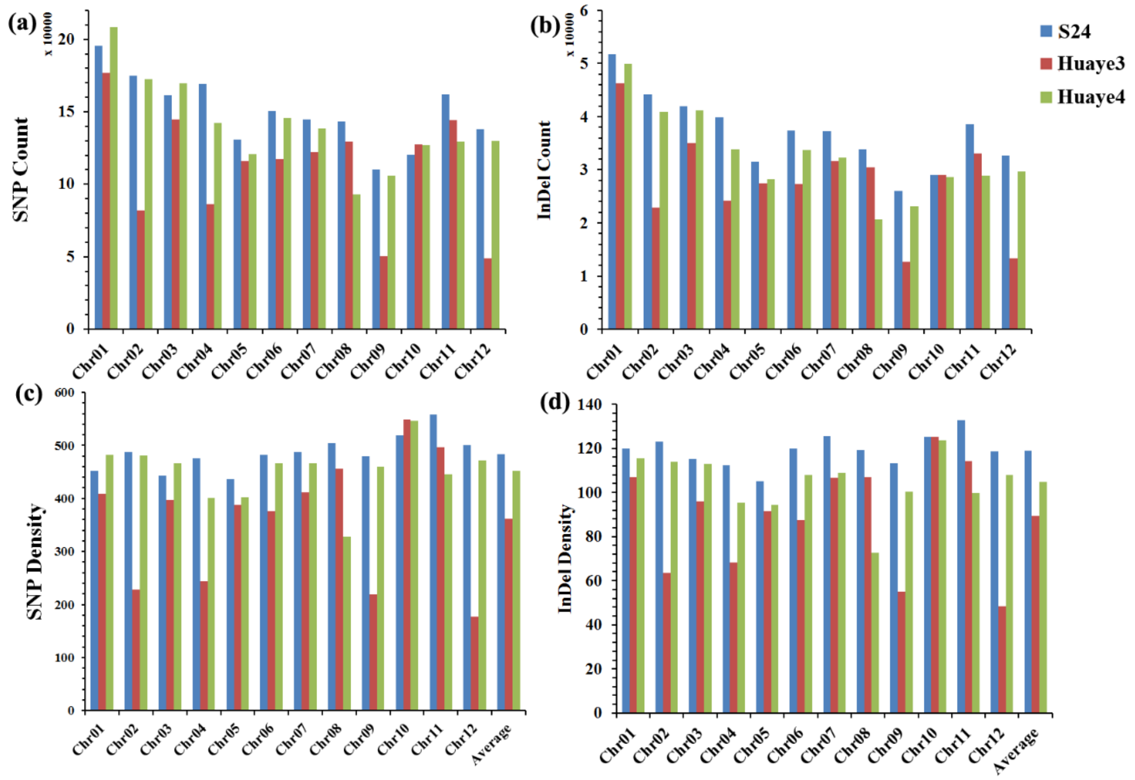

Fig.S4 Distribution of DNA polymorphisms number and density in common wild rice lines.

(a, b) Number of SNPs (a) and InDels (b) in different chromosomes. (c, d) Density of SNPs (c) and InDels (d) in different chromosomes.

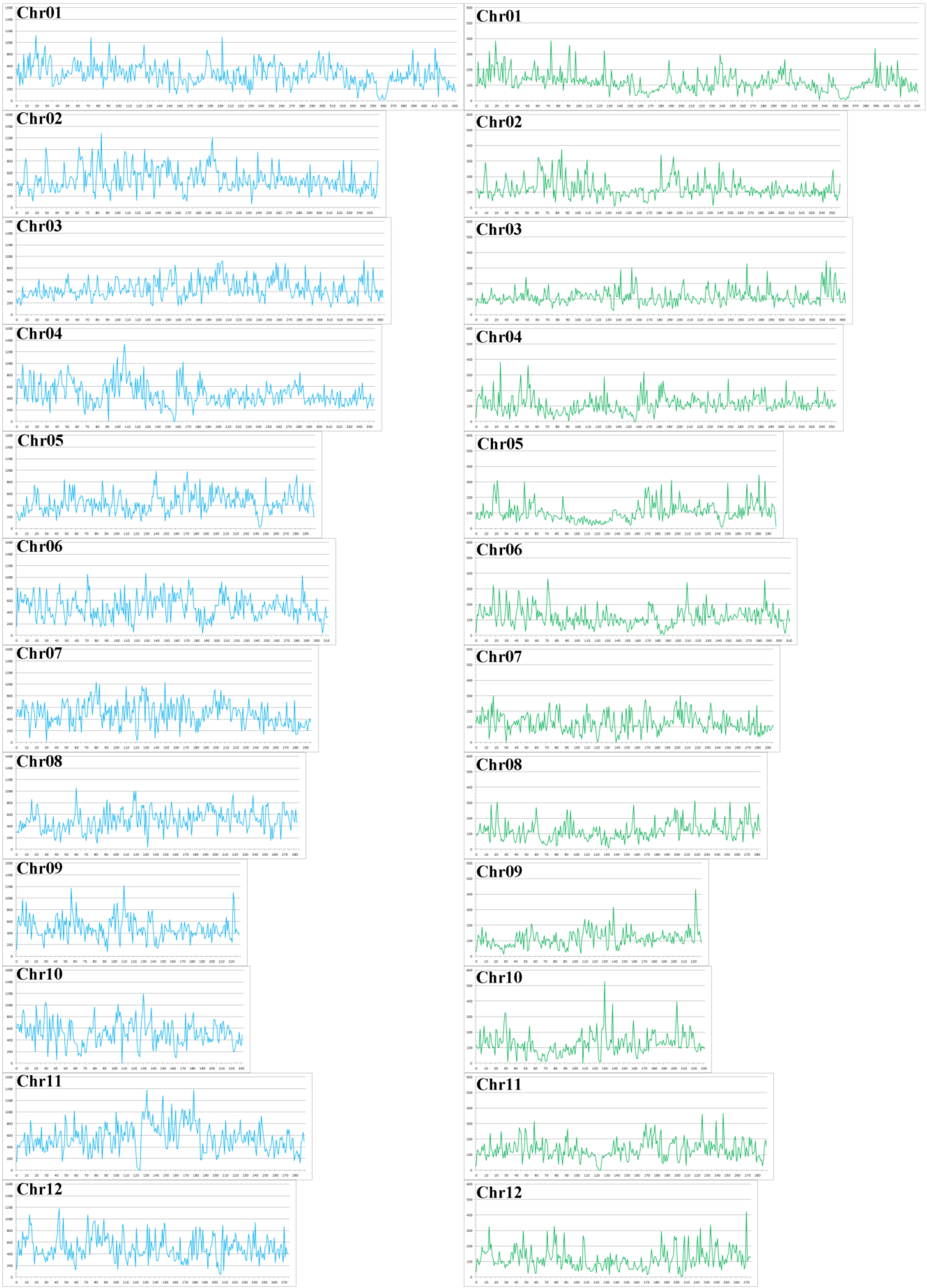

Fig.S5a Distribution of SNPs (blue lines) and InDels (green lines) in 100 kb windows across different chromosomes in S24.

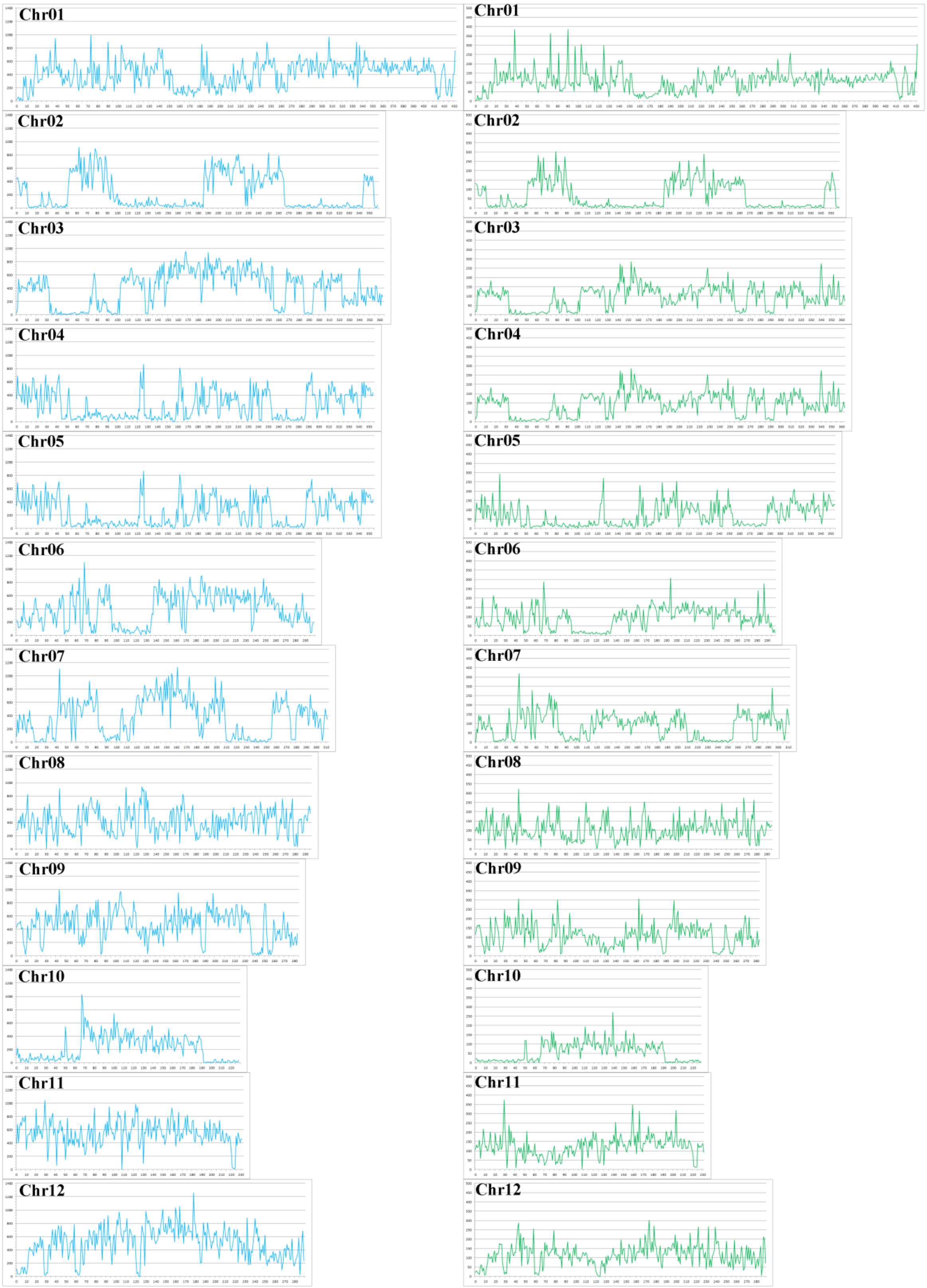

Fig.S5b Distribution of SNPs (blue lines) and InDels (green lines) in 100 kb windows across different chromosomes in Huaye 3.

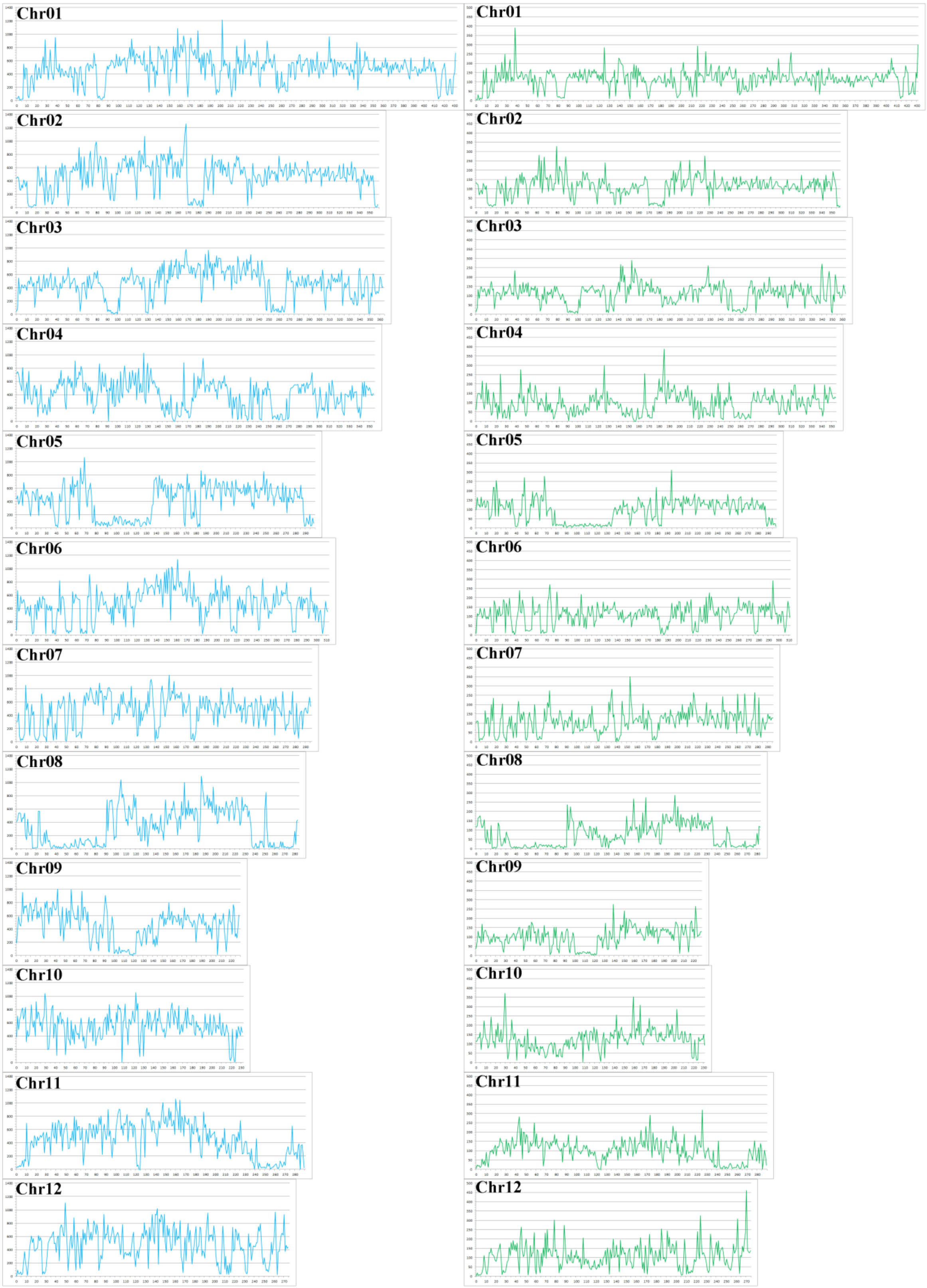

Fig.S5c Distribution of SNPs (blue lines) and InDels (green lines) in 100 kb windows across different chromosomes in Huaye 4.

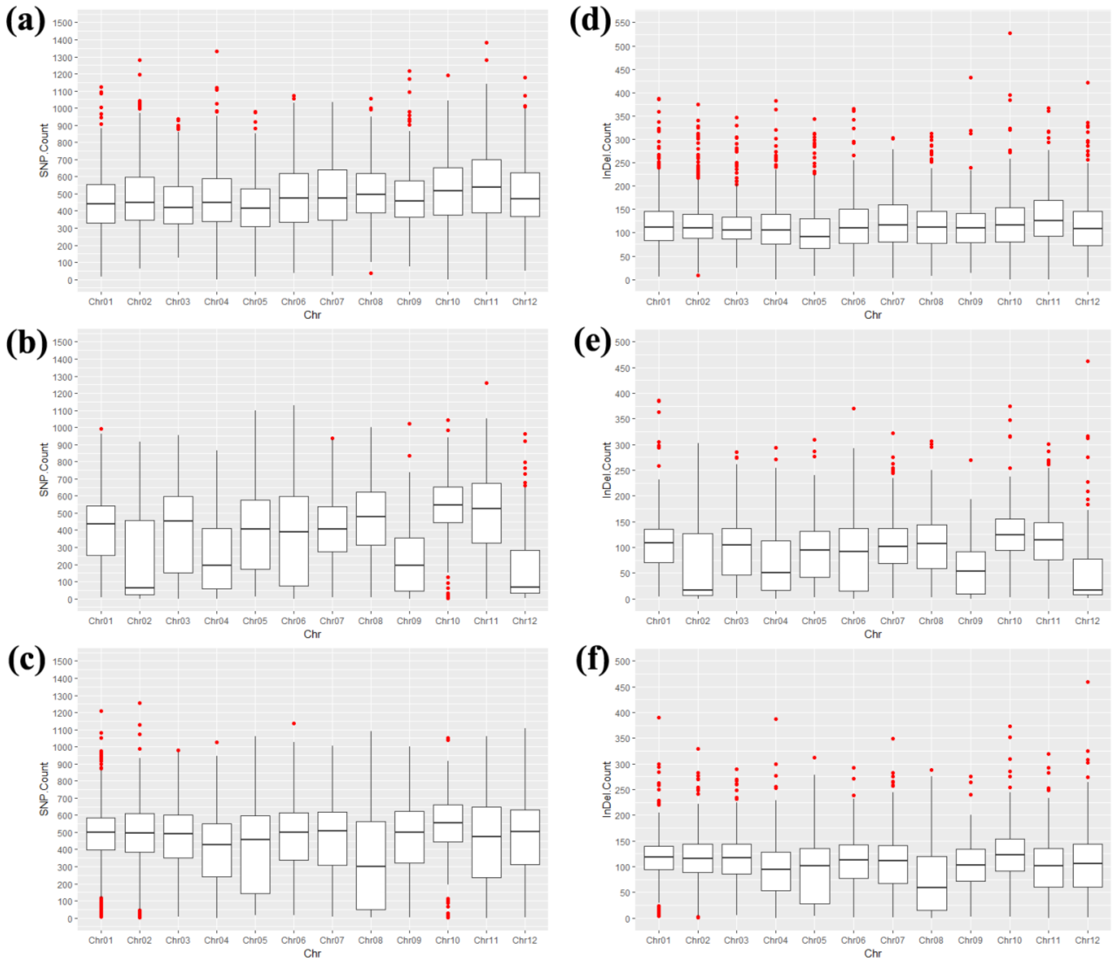

Fig.S6 Boxplot of DNA polymorphisms density in every 100 kb window.

(a, b, c) Boxplot of SNP density across the 12 chromosome in S24 (a), Huaye 3 (b) and Huaye 4 (c). (d, e, f) Boxplot of InDel density across the 12 chromosome in S24 (d), Huaye 3 (e) and Huaye 4 (f).

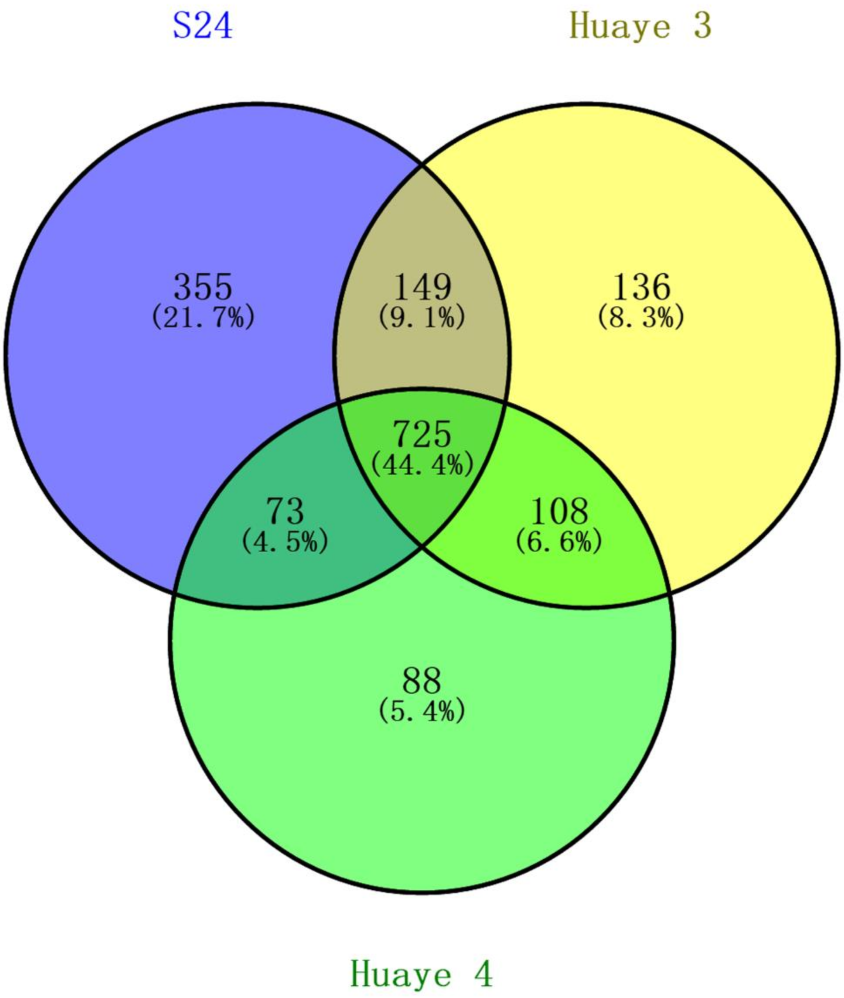

Fig.S7 Venn analysis of common wild rice unique genes detected in S24, Huaye 3 and Huaye 4.

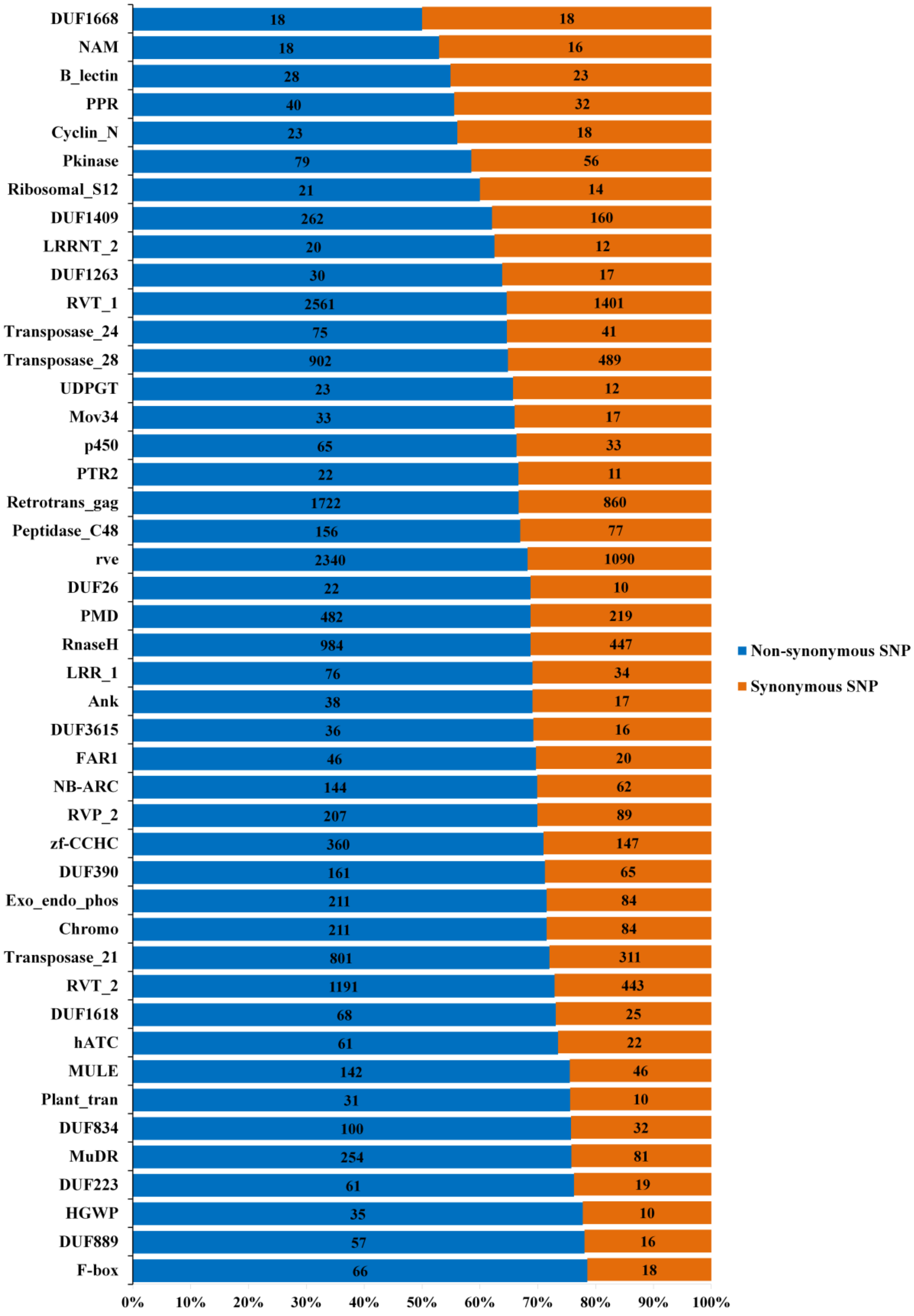

Fig.S8 Distribution of non-synonymous and synonymous SNPs in different Pfam domain families

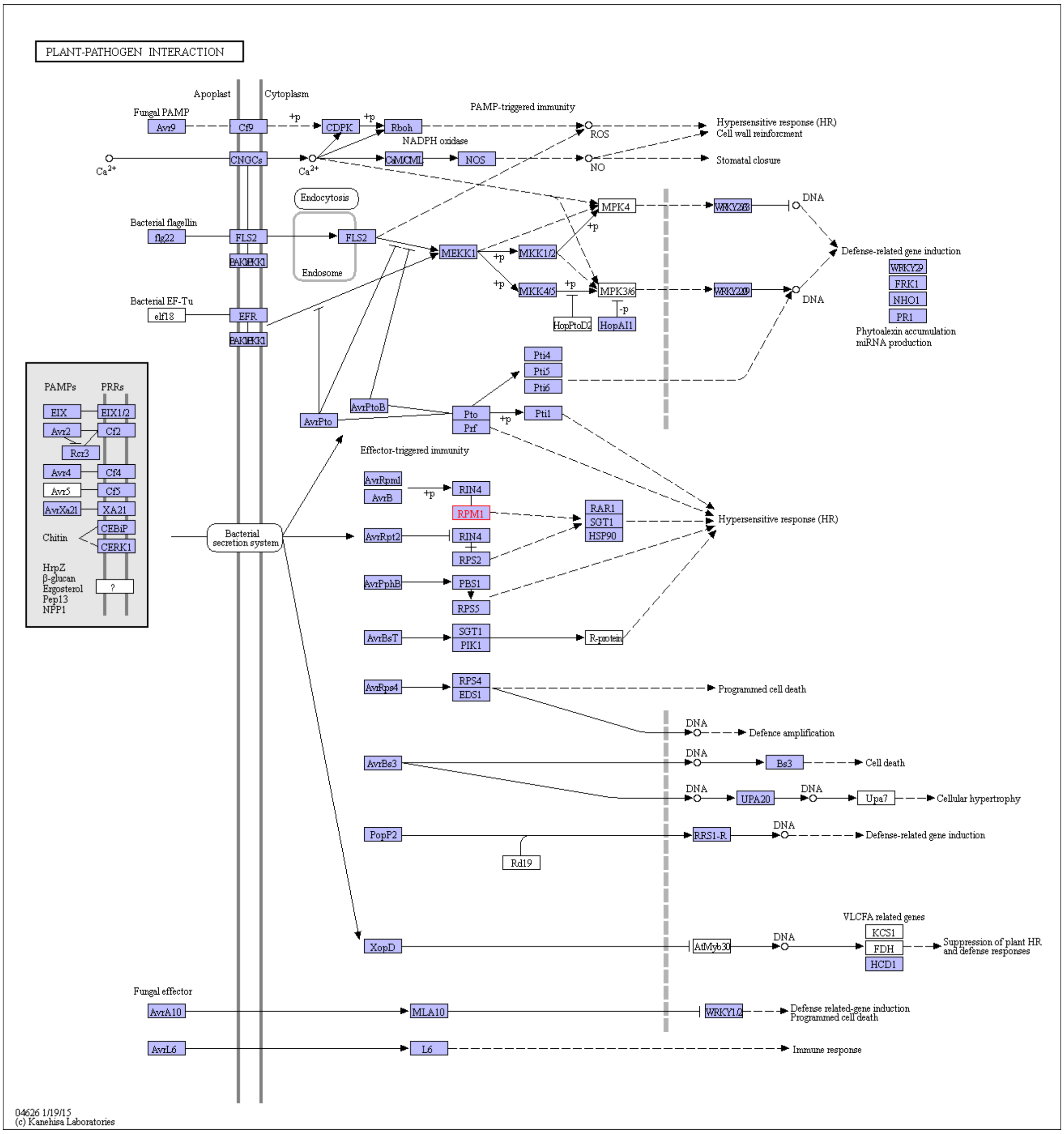

Fig.S9 KEGG pathway of a disease resistance protein RPM1 homologue. The RPM1 homologue was marked as red color.

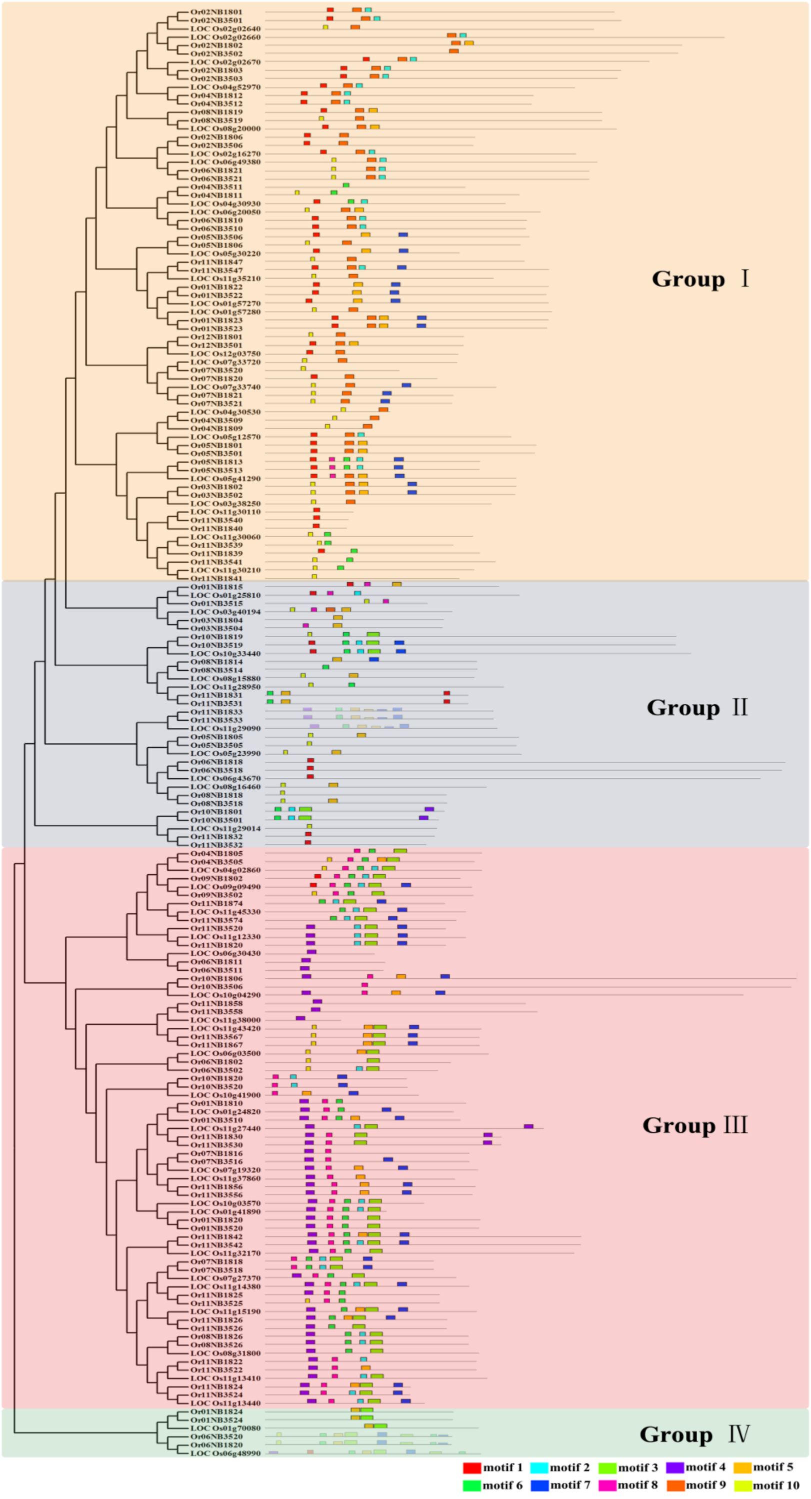

Fig.S10 Phylogenetic and MEME analysis of candidate genes and their homologs in the two inbred lines. The serial numbers of homologs in Huaye 3 and Huaye 4 were named as "Or-Chr-NB-18/35-No.". "Or" represents *Oryza rufipogon*, "Chr" represents chromosome, "NB" represents NB-ARC, "18" represents Huaye 3, "35" represents Huaye 4 and "No." represents serial number.

Motif 1 1.8e-810

Motif 2 2.3e-808

Motif 3 5.6e-1327

Motif 4 2.6e-1127

Motif 5 3.6e-952

Motif 6 1.9e-636

Motif 7 4.0e-759

Motif 8 5.0e-566

Motif 9 2.2e-884

Motif 10 1.1e-552

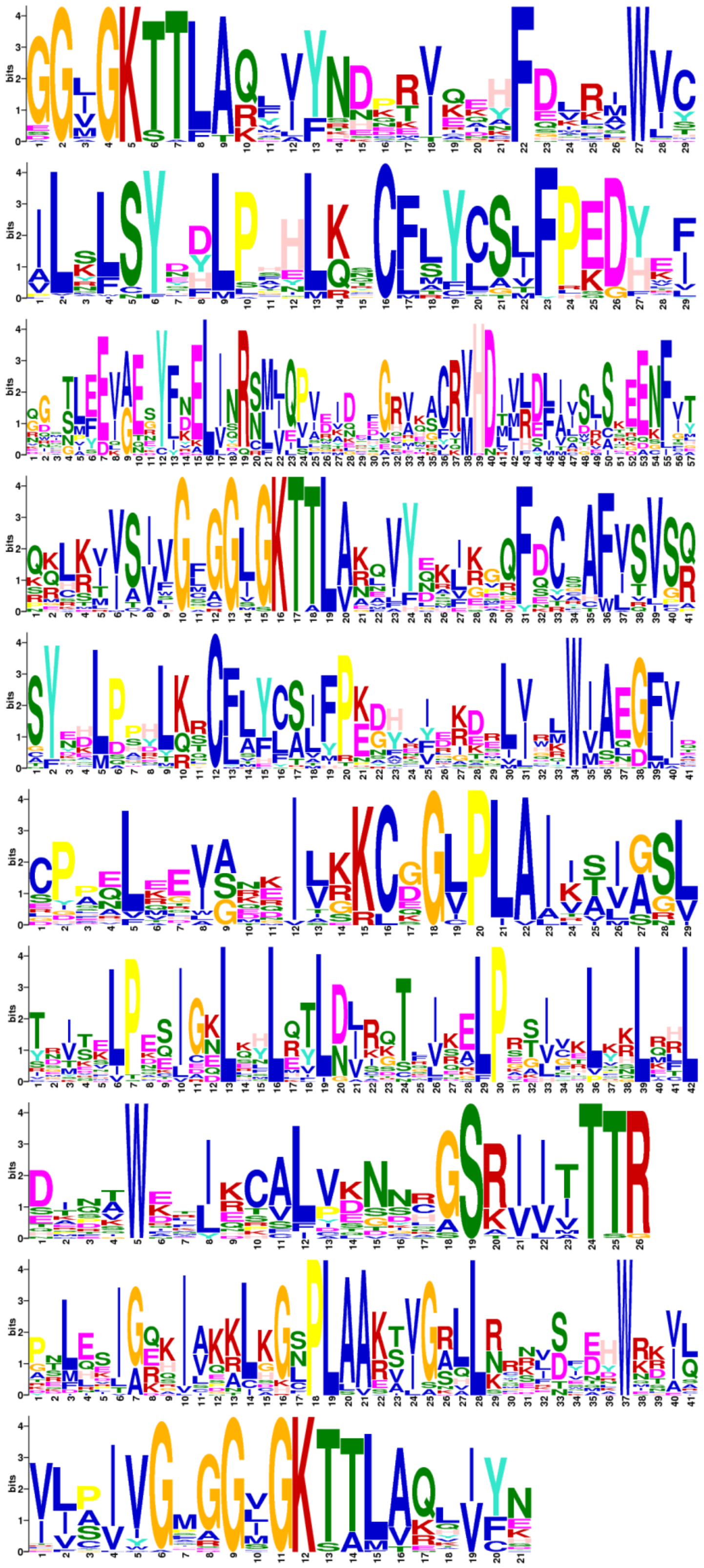

Fig.S11 Sequence logos of MEME motifs

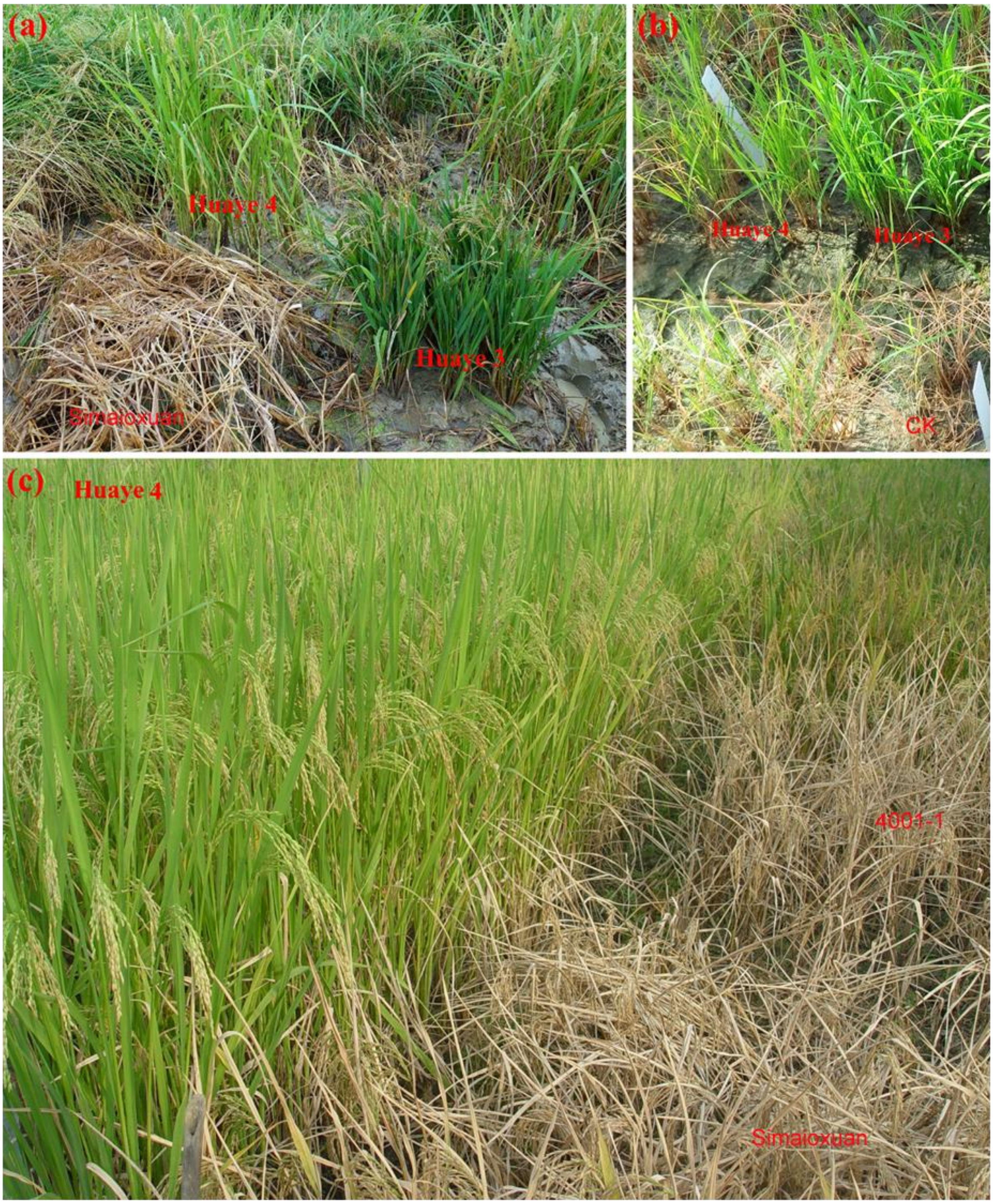

Fig.S12 Brown planthopper (BPH) resistance performance of Huaye 3 and Huaye 4.

(a) Resistance performance of Huaye 3 and Huaye 4 against BPH in epidemic year of 2017. Susceptible variety: Simiaoxuan. (b) The results of brown planthopper resistance assay of Huaye 3 by artificial inoculation. CK: Taichuang Native 1 (TN1). (c) Resistance performance of Huaye 4 against BPH in 2015. Susceptible varieties: Simiaoxuan and 4001-1.
